# Misoprostol Versus Oxytocin for the Prevention of Postpartum Haemorrhage: A Systematic Review and Meta‐Analysis Including Individual Participant Data

**DOI:** 10.1111/1471-0528.18197

**Published:** 2025-05-13

**Authors:** Madeline Flanagan, Nicole Au, Malitha Patabendige, Arsheeya Rattan, Ritwik Samanta, Daljit Sahota, Enrique Teran, Vanita Jain, Abdulkarim O. Musa, Munir'deen A. Ijaiya, Daniel L. Rolnik, Wentao Li, Ben W. Mol

**Affiliations:** ^1^ Department of Obstetrics and Gynaecology, Monash Medical Centre Monash University Clayton Victoria Australia; ^2^ Department of Obstetrics and Gynaecology Monash Health Melbourne Australia; ^3^ Department of Obstetrics and Gynaecology Kothari Medical Centre Kolkata West Bengal India; ^4^ Department of Obstetrics and Gynaecology Prince of Wales Hospital The Chinese University of Hong Kong Hong Kong SAR China; ^5^ Colegio de Ciencias de la Salud Universidad San Francisco de Quito Quito Ecuador; ^6^ Department of Obstetrics and Gynecology Postgraduate Institute of Medical Education and Research Chandigarh India; ^7^ Department of Obstetrics and Gynecology Federal Medical Centre Lokoja Nigeria; ^8^ Department of Obstetrics and Gynaecology, Faculty of Clinical Sciences University of Ilorin Ilorin Nigeria; ^9^ National Perinatal Epidemiology and Statistics Unit, Centre for Big Data Research in Health and School of Women's and Children's Health The University of New South Wales Sydney Australia; ^10^ Institute of Applied Health Sciences, Aberdeen Centre for Women's Health Research, School of Medicine, Medical Sciences and Nutrition University of Aberdeen Aberdeen UK

**Keywords:** active management of third‐stage labour (AMSTL), individual‐participant data meta‐analysis (IPD‐MA), meta‐analysis, misoprostol, oxytocin, postpartum haemorrhage (PPH), prevention

## Abstract

**Background:**

Postpartum haemorrhage (PPH) is the leading cause of maternal mortality. Uterotonics are the mainstay of PPH prevention.

**Objectives:**

To compare the efficacy of misoprostol and oxytocin for the prevention of PPH and to evaluate the trustworthiness of these randomised controlled trials (RCTs).

**Search Strategy and Selection Criteria:**

Seven databases were searched for peer‐reviewed literature meeting the inclusion criteria of RCTs comparing misoprostol and oxytocin for the prevention of PPH.

**Data Collection and Analysis:**

Data were collected by two independent reviewers. Individual participant data (IPD) were meta‐analysed for outcomes PPH ≥ 500 and ≥ 1000 mL. RCTs that did not share IPD were classified as trustworthy or not, and aggregate data were meta‐analysed according to trustworthiness.

**Main Results:**

Of 79 eligible RCTs, 10 (12.7%) provided IPD, of which 6 were included. Analysis of IPD showed PPH ≥ 500 mL to be significantly higher in the misoprostol than in the oxytocin group (2022 participants, aOR 1.84, 95% CI 1.43–2.34). For PPH ≥ 1000 mL, analysis of IPD showed that misoprostol and oxytocin were comparable (2022 participants, OR 1.14, 95% CI 0.68–1.91).

Of the 69 studies that did not provide IPD, 23 (33.3%) were assessed as trustworthy. Analysis of trustworthy data (IPD and 23 aggregate data RCTs) showed no difference between misoprostol and oxytocin for PPH ≥ 500 mL (24 334 participants, OR 1.01, 95% CI 0.69–1.49), while misoprostol was associated with a significantly increased risk of PPH ≥ 1000 mL compared to oxytocin (25 249 participants, OR 1.36, 95% CI 1.16–1.59).

**Conclusions:**

Of 79 RCTs comparing misoprostol and oxytocin for the prevention of PPH, 36.7% met trustworthiness criteria. Oxytocin is comparable to misoprostol for preventing PPH and may be superior for preventing severe PPH.

## 1 Introduction

Postpartum haemorrhage (PPH), defined as blood loss ≥ 500 mL within 24 h after birth, and severe PPH as blood loss of ≥ 1000 mL [1], is the leading global cause of maternal mortality and morbidity, resulting in 70 000 fatalities annually [2]. Uterine atony is the most common cause of PPH [3], the majority of PPH cases have no identifiable risk factors [4], rendering targeted prevention at an early stage difficult.

PPH prevention, or ‘active management of the third stage of labour (AMTSL)’, describes the two‐step process of administering a prophylactic uterotonic and delivering the placenta using controlled cord traction and uterine massage [1]. Administration of a uterotonic agent to the mother immediately after birth of the baby is the most important step of AMTSL. Oxytocin is the most used uterotonic due to its proven effectiveness, low incidence of maternal side effects and low cost. Other agents used individually, including ergometrine, carbetocin and misoprostol, that might be more effective, are associated with maternal side effects and/or are more expensive [5]. Oxytocin cannot be stored at temperatures above 15°C and therefore is not suitable for use in low‐resourced settings where cooled transport and storage are not feasible [6].

There are many data meta‐analyses comparing uterotonics for the prevention of PPH [1, 7]. In 2018, Cochrane published a landmark network meta‐analysis (NMA) evaluating the efficacy of all uterotonic agents used in the prevention of PPH [5] and concluded that misoprostol and oxytocin are similarly effective for the prevention of PPH ≥ 500 mL (relative risk [RR] 1.08, 95% confidence interval [CI] 0.94–1.24), and that misoprostol was associated with a significantly increased risk of PPH ≥ 1000 mL compared to oxytocin by 26% (RR 1.26, 95% CI 1.11–1.43).

Given recent increasing concerns about the trustworthiness of randomised controlled trials (RCTs) in women's health [8, 9], there is a need to evaluate the evidence base for uterotonic interventions upon which many current guidelines and clinical recommendations are based [10, 11, 12]. Individual participant data meta‐analysis (IPD‐MA) might meet this need as it provides an opportunity to test data trustworthiness from original investigators. IPD methodology also has the benefit of increased statistical power and the ability to adjust for prognostic factors and redefine and harmonise exposures and outcomes [13, 14, 15]. To our knowledge, this is the first meta‐analysis to interrogate the trustworthiness of IPD and aggregate data comparing misoprostol and oxytocin for the prevention of PPH.

## 2 Objectives

This IPD MA aimed to compare the efficacy and safety of misoprostol and oxytocin for the prevention of PPH. The aggregate data MA aimed to pool data from trustworthy RCTs and compare the results to those generated from untrustworthy ones. By doing so, we will understand how the trustworthiness and quality of the RCTs affect estimates comparing misoprostol and oxytocin for the prevention of PPH.

## 3 Materials and Methods

This international collaborative IPD‐MA followed a prospectively registered protocol (PROSPERO: CRD42022343340) and a statistical analysis plan produced in advance of data lock and analysis (publicly available from: https://www.crd.york.ac.uk/prospero/). Ethics approval was obtained from the Monash University Human Research Ethics Committee on 13/7/2022 (Project ID#34839). Findings are reported following the Preferred Reporting Items for Systematic reviews and Meta‐analyses (PRISMA)‐IPD reporting guideline (Appendix A and B) [16].

### 3.1 Search Strategy

Relevant RCTs (those comparing misoprostol vs. oxytocin) from the 2018 Cochrane NMA comparing uterotonics for the prevention of PPH [5] were included. A list of all RCTs included or designated ‘awaiting classification’ in the 2018 Cochrane NMA was reviewed. RCTs comparing misoprostol and oxytocin were identified and included in this study. We independently developed a search strategy to search for RCTs published in the years following the 2018 Cochrane NMA, from 2018 to May 30th, 2023. We updated our search on October 22nd, 2024. Ovid MEDLINE, Ovid Embase, Ovid Emcare, CINAHL Plus, Scopus, Cochrane Central Register of Controlled Trials, clinicaltrials.gov and reference lists of retrieved studies were searched to identify further published RCTs (search strategy outlined in Appendices C–F). Two investigators (M.F. and A.R.) independently reviewed the identified titles and abstracts, followed by the full texts for eligibility, with disagreements resolved by a third reviewer (M.P.).

### 3.2 Eligibility Criteria

All RCTs comparing systemically administered misoprostol (any dose) and oxytocin (any dose) were included. Studies with other designs, including cluster RCTs and quasi‐experimental designs, were excluded. There were no language restrictions, and all published and unpublished data were eligible. If an identified study was published in an unfamiliar language to the research team, Google Translate was used to translate the study into English. Professional translation services were sought if further translation or clarification was required.

### 3.3 Data Access

We approached investigators of eligible RCTs to share IPD. Trial investigators' contact details were obtained through the published articles or their institutional websites. IPD‐MA invitations were emailed at least four times if there was no response. Where the primary or corresponding authors' contact details were unavailable or no response was obtained, attempts were made to contact other authors involved in the RCTs, and lead/co‐authors were copied in at least every month. If authors were not responding to e‐mail, other contact details were sought from institutional affiliations and social media platforms. Our academic contacts in particular countries were also used to contact the authors and/or their institutions who were not responding to the initial enquiries. Journal editors were contacted as a last resort for some studies.

RCT investigators who agreed to partake in this study supplied de‐identified IPD, which were harmonised and recoded to the pre‐defined IPD meta‐analysis definitions. Data were requested for all randomised participants, even if excluded from the original trial analyses. The received data were examined for missing data, error, internal consistency, consistency with the publication and pattern of treatment allocation and data presentation, where possible [17]. Identified issues were communicated with RCT investigators for a solution before acceptance in the IPD meta‐analysis dataset. Studies with unresolvable inconsistencies or trustworthiness concerns were excluded from the analysis.

### 3.4 Description of the Intervention

Misoprostol and oxytocin are uterotonic medications administered during the third stage of labour after delivery of the baby, prior to delivery of the placenta. Misoprostol can be delivered via different routes including oral/per os (PO), buccal, sublingual (SL), per vaginal (PV) and per rectal (PR). Common doses of misoprostol range from 200 to 1000 μg. Oxytocin may be administered intravenously (IV) or intramuscularly (IM). Common doses of oxytocin range from 5 to 20 IU.

### 3.5 Outcomes and Effect Modifiers

The primary outcomes included PPH ≥ 500 and ≥ 1000 mL. Estimated blood loss (EBL) was used to determine the occurrence of PPH ≥ 500 and ≥ 1000 mL. Trialists EBL through collection and gravimetric measurement, or visual estimation. The method of EBL measurement is outlined in Appendix H. Secondary outcomes included EBL, the difference between haemoglobin change (Hb drop; g/dL), the difference between haematocrit change (HCT; %), additional uterotonic use and blood transfusion requirement, and participant side effects, including headache, nausea, vomiting, diarrhoea, shivering and pyrexia (Table S1). Potential effect modifiers of interest for PPH included parity, participant age and gestational age at delivery. Subgroup analysis was performed according to BMI and pre‐delivery Hb because these are well known prognostic factors of PPH [18, 19]. As sensitivity analysis in vaginal mode of delivery (MOD) was planned.

### 3.6 Risk of Bias, Certainty and Trustworthiness of Included RCTs

The risk of bias (RoB) was assessed by one reviewer (M.F.) using the Cochrane RoB‐2 tool [20]. Each study was assessed individually. These scores were compared to the RoB‐2 scores from the 2018 Cochrane NMA [5]. Differences were resolved by a third reviewer (M.P.). If the information was insufficient, clarification was sought from trialists. The Trustworthiness in Randomised Controlled Trials (TRACT) tool [21] was used to screen RCTs for data integrity concerns (Appendix G). The TRACT tool evaluates seven domains, including: governance, author group, plausibility of intervention usage, timeframe, drop‐out rates, baseline characteristics and outcomes, to determine whether an RCT has concerns for data integrity. One reviewer (M.F.) independently screened all RCTs and categorised them as ‘low’, ‘moderate’ or ‘high’ risk for data integrity concerns. Moderate‐ and high‐risk studies were confirmed by a second reviewer (B.W.M.). The GRADE tool was applied by two reviewers (M.F. and M.P.).

For studies where IPD were obtained [22, 23, 24, 25, 26, 27], we assessed integrity using an IPD‐integrity tool [17]. Our definition of ‘trustworthy data’ included IPD after data checks and RCTs that were assessed as low‐risk with the TRACT tool. Our definition of data ‘not meeting trustworthiness criteria’ was IPD excluded after data checks, and RCTs assessed as moderate‐ and high‐risk with the TRACT tool.

### 3.7 Data Synthesis

For each outcome, an intention‐to‐treat analysis was performed using all available data comparing misoprostol to oxytocin. An as‐treated sensitivity analysis was planned. In this IPD meta‐analysis, oxytocin was considered the reference group for all outcomes.

The primary analysis strategy was a one‐stage meta‐analysis method to synthesise the IPD. One stage was necessary as some data reported a low occurrence of the primary outcomes. For the one‐stage method, we used multilevel mixed‐effects logistic regression (a stratified intercept by study and a random treatment effect, covariates as fixed effects, maximum likelihood estimator), adjusting for participant age, parity and gestational age [28]. We explored treatment‐covariate interactions for PPH using interaction terms between treatment and potential effect modifiers, adjusting for the added main effects of participant age, parity and gestational age for each included study. Only within‐trial interaction was considered to avoid ecological bias. Binary outcomes were reported as odds ratio (OR) and continuous outcomes as mean difference (MD).

All variables were checked for missing values and entries outside the expected ranges. Variables that were missing > 0.01% of observations were analysed separately for each dataset using the patterns chart of missing data. In the event of missing values for covariates or potential effect modifiers in any RCT, we performed multiple imputations using chained equations within the RCT before the meta‐analysis.

Aggregate data meta‐analysis was planned for trials that did not share IPD. Summary statistics were extracted from trial publications, and a two‐stage meta‐analysis using a random effects model was performed. One MA included all RCTs regardless of IPD sharing. The second MA included an assessment of trustworthiness, comparing RCTs and IPD classified as ‘trustworthy’ to RCTs and IPD that did not satisfy trustworthy criteria. In line with the primary analysis, the primary outcomes for the aggregate data meta‐analysis were PPH ≥ 500 and ≥ 1000 mL. Secondary outcomes were analysed if sufficient summary statistics were available.

Statistical analyses were performed using the R statistical environment (R Foundation for Statistical Computing, Vienna, Austria) [29]: ‘meta’ and ‘lme4’ packages were used for the summary statistics, one‐stage meta‐analysis, and aggregate data meta‐analysis.

## 4 Results

### 4.1 Study Selection and Participants

**FIGURE 1:**
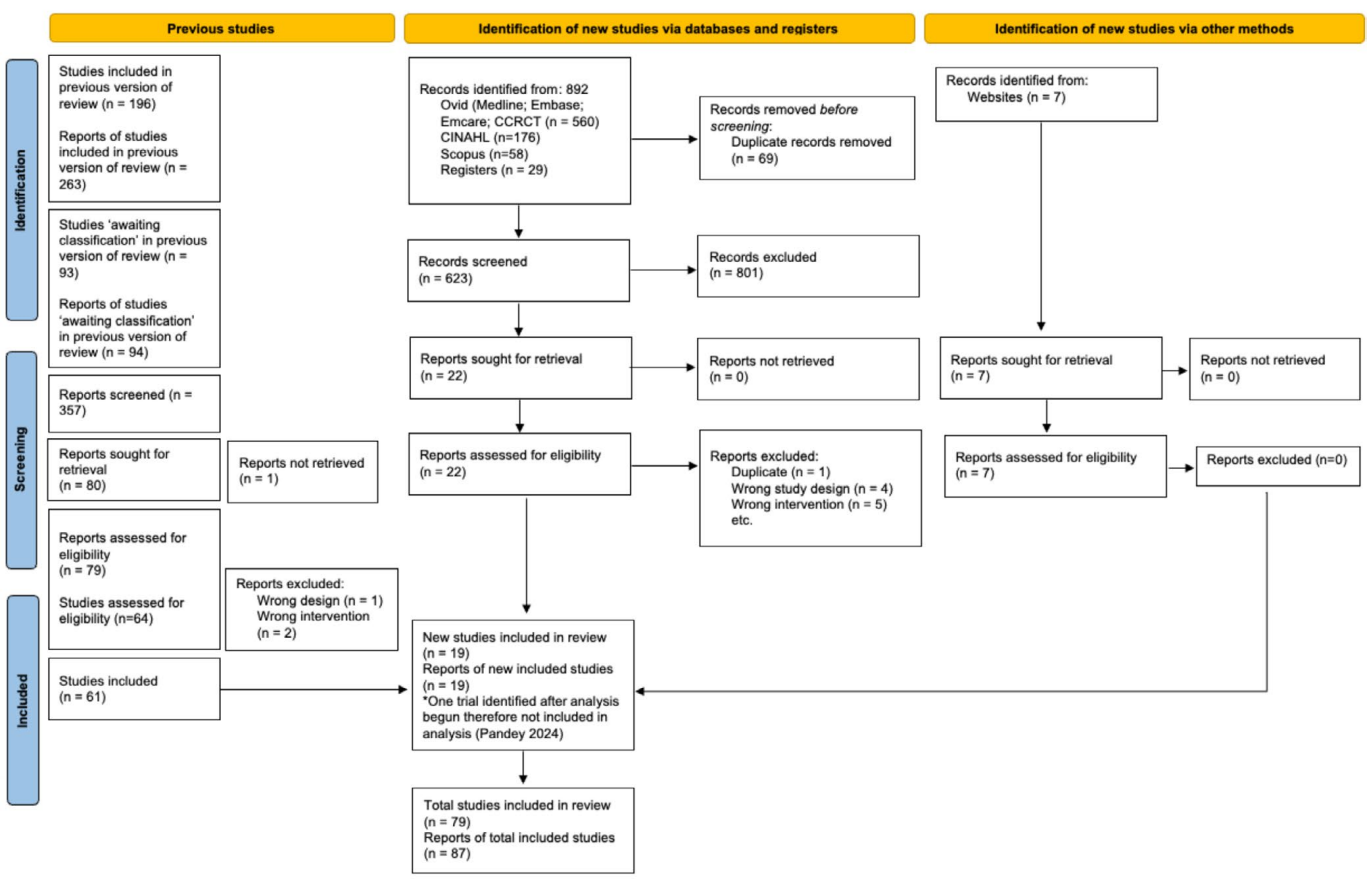
Trial identification (PRISMA‐IPD flow diagram [16]). PRISMA: Preferred Reporting Items for Systematic Reviews and Meta‐Analyses.

RCTs designated ‘awaiting classification’ status and RCTs included in the 2018 Cochrane NMA were screened (*n* = 289); 61 were included. Our additional search identified 892 unique references published since 2018. After abstract and full text screening, 12 RCTs were included. A further 7 RCTs were identified during the screening process, totalling 80 RCTs. One RCT [30] (*n* = 120) was identified in October 2024 after completion of data analysis and was not included. Our final study selection analysis included 79 RCTs and 44 941 participants, of which 6 RCTs with 2022 participants contributed IPD (PRISMA‐IPD flow diagram, Figure 1).

Of the 79 trial publications, authors of 31 RCTs (39.2%) did not respond to our invitation, and authors of 48 RCTs responded. Ten (12.7%) agreed to participate, while 38 (48.1%) declined. An overview of eligible RCTs, author responses, and reasons for declining participation is outlined in Table S2.

### 4.2 Study Characteristics

Of the 10 studies for which IPD were obtained, we excluded 4 after data checks. Reasons for excluding studies included missing data [31], concerns for randomization [32, 33], irreproducible baseline characteristics and results [33], and concerns for data quality [34]. All authors responded to our e‐mail enquiries, but our concerns were unresolved, so these data were excluded from IPD‐MA (Table S3). The final IPD analysis included six RCTs, totalling 2022 participants (Table 1) [22, 23, 24, 25, 26, 27].

**TABLE 1:** Characteristics of included trials.

#	Author	Year	Country	Misoprostol	Oxytocin	Inclusion criteria	# Participants in RCT	# Cases used in MA
1	Atukunda [22]	2014	Uganda	600 μg SL	10 IU IM	Age ≥ 18 years, 38–41 weeks of amenorrhoea, anticipated uncomplicated vaginal delivery	1140	1140
2	Burman [23]	2021	India	600 μg PR	10 IU IM	37–41 weeks' gestation, patients in active labour	80	80
3	Ng/Daljit [24]	2004	Hong Kong	400 μg PO	10 IU IV	Singleton pregnancy, vaginal delivery	309	302
4	Gavilanes [27]	2016	Ecuador	100 μg SL	10 IU IV	All women undergoing elective CS	100	100
5	Gupta [26]	2006	India	600 μg PR	10 IU IM	Women in spontaneous labour anticipated vaginal delivery	200	200
6	Musa [25]	2015	Nigeria	600 μg PR	10 IU IM	15–42 years old, term, advanced first stage labour, having spontaneous vaginal delivery, no or low risk of PPH	200	200

Abbreviations: CS: caesarean section; IM: intra‐muscular; IV: intravenous; PO: per‐os (by mouth); PPH: postpartum haemorrhage; PR: per‐rectal; SL: sublingual.

### 4.3 RoB in Included Studies

On screening for RoB with the RoB‐2 tool [20], 75 of 79 (94.9%) RCTs were identified as having ‘some concerns’, or being ‘high risk’ for bias. Seventy‐one trials (89.9%) had ‘some concerns’ for *selective reporting bias*, due to the unavailability of a prespecified study protocol or statistical analysis plan (SAP; Figures S1 and S2). Thirty‐nine trials (49.4%) were ‘high‐risk’ for *objective measurement of outcome*, as they used visual estimation of blood loss by assessors who were not blinded to patient treatment allocation.

### 4.4 Screening of All RCTs With the TRACT Tool

Sixty‐nine RCTs that did not contribute IPD were screened with the TRACT tool (Table S2). Twenty‐three (33.3%) RCTs were identified as low‐risk, 39 (56.5%) were moderate‐risk and 7 (10.1%) were high‐risk for trustworthiness concerns.

### 4.5 Descriptive Analysis of Participants

Of the 2022 participants for whom we received IPD, 1012 (50.0%) were allocated to misoprostol and 1010 (50.0%) to oxytocin. The average participant age in the oxytocin group was 27.2 ± 5.0 years, and in the misoprostol group, it was 27.2 ± 5.3 years. In the oxytocin group, 59% of participants were multiparous and 41% were nulliparous, compared with 56% multiparity and 44% nulliparity in the misoprostol group. The average gestational age was 39.13 ± 1.27 weeks in the oxytocin group and 39.16 ± 1.23 weeks in the misoprostol group. Baseline participant characteristics are available in Table S4.

### 4.6 Synthesis of Results

#### 4.6.1 Primary Outcomes: IPD‐MA

**FIGURE 2:**
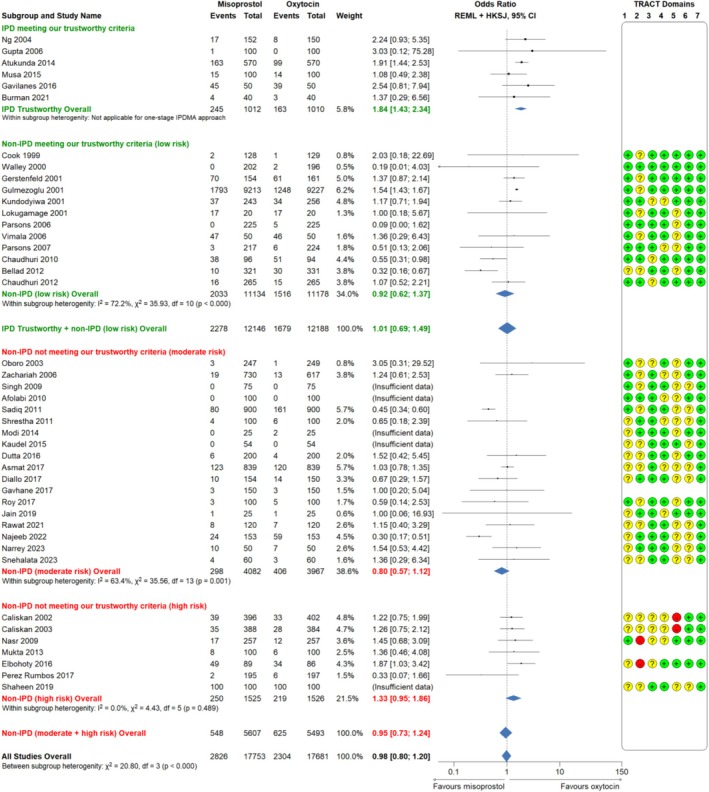
Misoprostol compared to oxytocin for the outcome PPH ≥ 500 mL; forest plot comparison of IPD MA and aggregate data MA of RCTs *low, moderate and high risk* for data integrity concerns (according to TRACT assessment). HKSJ: Hartung and Knapp, Sidik and Jonkman (method of estimation for confidence intervals); IPD: Independent participant data; REML: Restricted maximum likelihood (overall treatment effect estimation); WHO: World Health Organisation.

**FIGURE 3:**
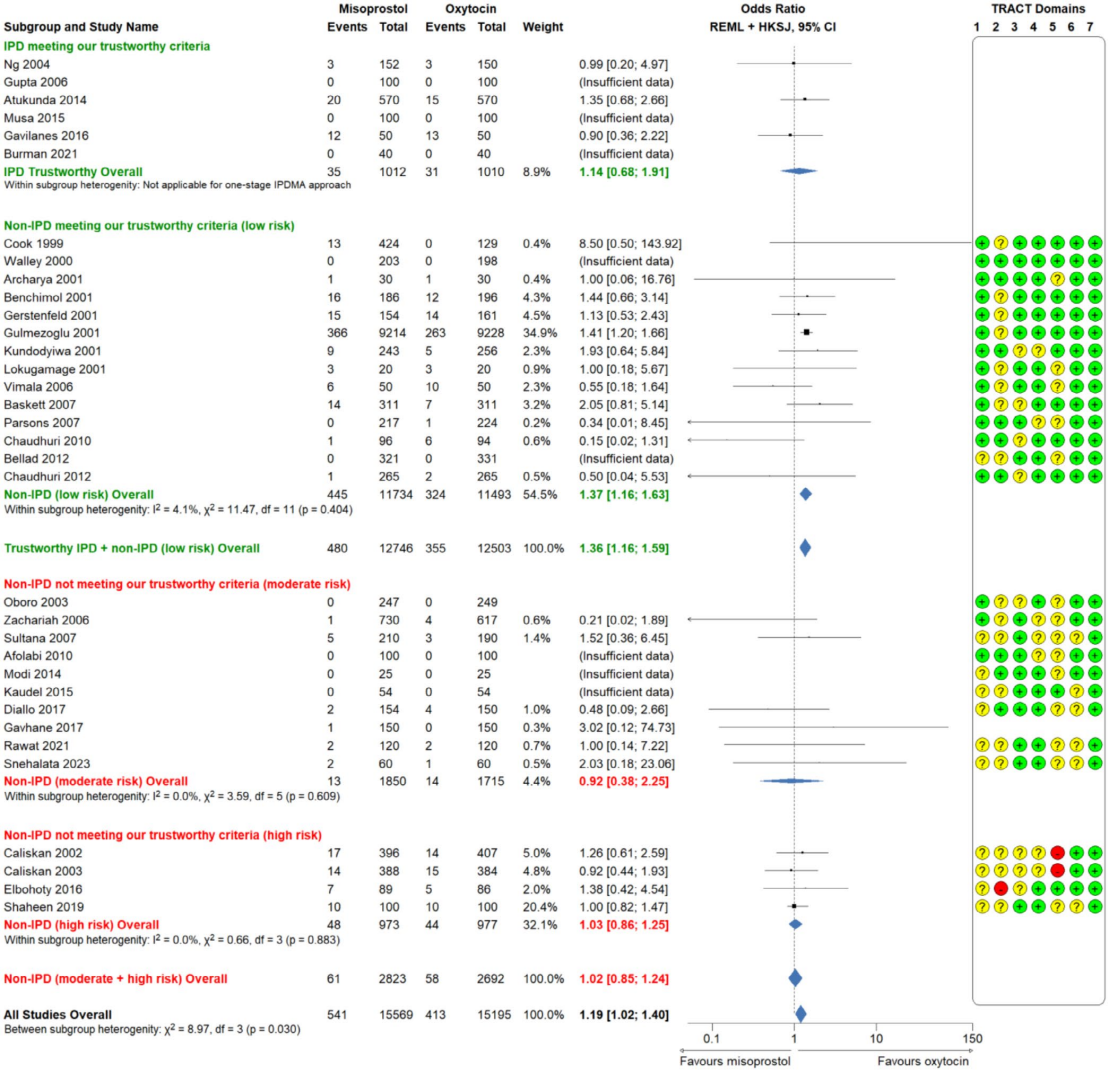
Misoprostol compared to oxytocin for the outcome PPH ≥ 1000 mL; forest plot comparison of IPD MA and aggregate data MA of RCTs *low, moderate and high risk* for data integrity concerns (according to TRACT assessment). HKSJ: Hartung and Knapp, Sidik and Jonkman (method of estimation for confidence intervals); IPD: Independent participant data; REML: Restricted maximum likelihood (overall treatment effect estimation); WHO: World Health Organisation.

Misoprostol was associated with a significant increase in PPH ≥ 500 mL (6 RCTs, 2022 participants, 24.2% vs. 16.1%, aOR 1.84, 95% CI 1.43–2.34, *p* < 0.001; Figure 2, moderate certainty evidence, Tables S5 and S8). Misoprostol was associated with a non‐significant increase in PPH ≥ 1000 mL (6 RCTs, 2022 participants, 3.5% vs. 3.1%, aOR 1.14, 95% CI 0.68–1.91, *p* = 0.626; Figure 3, low certainty evidence, Tables S5 and S8).

#### 4.6.2 Secondary Outcomes: IPD‐MA

The results for secondary outcomes can be seen in Table S5. Misoprostol use was associated with a significant increase in mean EBL (6 RCTs, 2022 participants, misoprostol mean EBL 425.1 mL [SD = 244.6 mL]; oxytocin mean EBL 382.5 mL [SD = 257.4 mL], aMD 41.18, 95% CI 21.00–59.14 mL, *p* < 0.001) and a non‐significant increase in mean haemoglobin change (5 RCTs, 1902 participants, aMD 0.08, 95% CI 0.00–0.11, *p* = 0.043) and additional uterotonic use (4 RCTs 1640 participants, aOR 1.30, 95% CI 0.92–1.85, *p* = 0.143). Misoprostol use was associated with a non‐significant lower rate of blood transfusion (4 RCTs, 1640 participants, aOR 0.43, 95% CI 0.18–1.06, *p* = 0.066). In terms of maternal side effects, misoprostol use was associated with a significant increase in vomiting (6 RCTs, 2010 participants, aOR 2.16, 95% CI 1.09–4.25, *p* = 0.027), diarrhoea (5 RCTs, 1904 participants aOR 3.97, 95% CI 1.52–10.37, *p* = 0.05), shivering (5 RCTs, 1811 participants, aOR 5.69, 95% CI 2.35–13.81, *p* < 0.001) and fever (3 RCTs, 693 participants, aOR 5.89, 95% CI 2.19–15.86, *p* < 0.001).

#### 4.6.3 Subgroup Analysis: IPD‐MA

The results of the subgroup analysis can be seen in Table S6. Misoprostol use was associated with a significantly increased risk of PPH ≥ 500 mL in both BMI < 25 kg/m^2^ (3 RCTs, 570 participants, aOR 1.94, 95% CI 1.26–2.98, *p* = 0.003) and BMI ≥ 25 kg/m^2^ (3 RCTs, 850 participants, aOR 1.88, 95% CI 1.30–2.72, *p* < 0.001) subgroups. Misoprostol use was associated with a non‐significant increase in PPH ≥ 1000 mL in participants with BMI < 25 kg/m^2^ (3 RCTs, 570 participants, aOR 2.41, 95% CI 0.62–9.37, *p* = 0.203) and ≥ 25 kg/m^2^ (3 RCTs, 850 participants, aOR 2.41, 95% CI 0.62–9.37, *p* = 0.203).

In anaemic participants, misoprostol use was associated with a non‐significant increase in PPH ≥ 500 mL (5 RCTs, 333 participants, aOR 1.31, 95% CI 0.59–2.87, *p* = 0.507) and PPH ≥ 1000 mL (5 RCTs, 333 participants, aOR 0.91, 95% CI 0.91–41.91, *p* = 0.963). In non‐anaemic participants, misoprostol was associated with a significantly increased risk of PPH ≥ 500 mL (5 RCTs, 1582 participants, aOR 1.90, 95% CI 1.46–2.49, *p* < 0.001) and a non‐significant increase in PPH ≥ 1000 mL (5 RCTs, 1582 participants, aOR 1.39, 95% CI 0.72–2.67, *p* = 0.331).

#### 4.6.4 Intervention‐Covariate Interaction

No differential impact of treatment on the rate of PPH ≥ 500 or ≥ 1000 mL was identified for participant multiparity, participant age (dichotomised as 18–35 and > 35), or gestational age (Table S7).

#### 4.6.5 Integration of IPD and Aggregate Data MA for Primary Outcomes

Of 69 RCTs that did not provide IPD, 23 RCTs (33.3%) were assessed as low risk for data integrity concerns. Of the 23 low‐risk RCTs, 12 reported the outcome PPH ≥ 500 mL, and 14 reported the outcome PPH ≥ 1000 mL. Thirty‐nine RCTs (56.5%) were assessed as moderate risk; 18 of these reported the outcome PPH ≥ 500 mL, and 10 reported PPH ≥ 1000 mL. Seven RCTs (10.1%) were assessed as high risk; five of these reported the outcome PPH ≥ 500 mL, and four reported the outcome PPH ≥ 1000 mL.

Regarding the outcome PPH ≥ 500 mL, analysis of trustworthy data showed misoprostol had similar efficacy to oxytocin (18 RCTs, 24 334 participants, OR 1.01, 95% CI 0.69–1.49, very low certainty evidence, Tables 2 and S8, Figure 2). Analysis of data not meeting our trustworthiness criteria showed misoprostol was associated with a non‐significant decrease in PPH ≥ 500 mL (25 RCTs, 11 100 participants, OR 0.95, 95% CI 0.73–1.24).

**TABLE 2:** Comparison of IPD‐MA and aggregate data MA (according to TRACT assessment): Misoprostol compared to oxytocin for the primary outcomes PPH ≥ 500 and ≥ 1000 mL.

Data type	# Trials	# Participants	Crude incidence: misoprostol vs. oxytocin *n*/*N* (%)	OR (95% CI)	Type of analysis	Heterogeneity
PPH ≥ 500 mL						
IPD	6	2022	Miso: 245/1012 (24.2)	1.84 (1.43; 2.34)^a^	One‐stage	*p* < 0.001**
			Oxy: 163/1010 (16.1)			
TRACT assessment: *Low risk*	12	22 312	Miso: 2033/11 134 (18.3)	0.92 (0.62; 1.37)	Two‐stage	*I* ^2^ = 72.2%, *χ* ^2^ = 35.93, df = 10 (*p* < 0.001)
			Oxy: 1516/11 178 (13.6)			
TRACT assessment: *Moderate risk*	18	8049	Miso: 298/4082 (7.3)	0.80 (0.57; 1.12)	Two‐stage	*I* ^2^ = 63.4%, *χ* ^2^ = 35.56, df = 13 (*p* = 0.001)
			Oxy: 406/3967 (10.2)			
TRACT assessment: *High risk*	7	3051	Miso: 250/1525 (16.4)	1.33 (0.95; 1.86)	Two‐stage	*I* ^2^ = 0.00%, *χ* ^2^ = 4.43, df = 5 (*p* = 0.489)
			Oxy: 219/1526 (14.4)			
Trustworthy data (IPD+*low‐risk* RCTs)	18	24 334	Miso: 2278/12 146 (18.8)	1.01 (0.69; 1.49)	Two‐stage	—
			Oxy: 1679/12 188 (13.8)			
Data not meeting trustworthy criteria (*moderate+high risk* RCTs)	25	11 100	Miso: 548/5607 (9.8)	0.95 (0.73; 1.24)	Two‐stage	—
			Oxy: 625/5493 (11.4)			
All data: IPD+aggregate data (*low/moderate/high risk* RCTs)	43	35 434	Miso: 2826/17 753 (15.9)	0.98 (0.80; 1.20)	Two‐stage	*χ* ^2^ = 20.80, df = 3 (*p* < 0.001)
			Oxy: 2304/17 681 (13.0)			
PPH ≥ 1000 mL						
IPD	6	2022	Miso: 35/1012 (3.5)	1.14 (0.68; 1.91)^a^	One‐stage	*p* = 0.626**
			Oxy: 31/1010 (3.1)			
TRACT assessment: *Low risk*	14	23 227	Miso: 445/11 734 (3.8)	1.37 (1.16; 1.63)	Two‐stage	*I* ^2^ = 4.1%, *χ* ^2^ = 11.47, df = 11 (*p* = 0.404)
			Oxy: 324/11 493 (2.8)			
TRACT assessment: *Moderate risk*	10	3565	Miso: 13/1850 (0.7)	0.92 (0.38; 2.25)	Two‐stage	*I* ^2^ = 0.0%, *χ* ^2^ = 3.59, df = 3 (*p* = 0.609)
			Oxy: 14/1715 (0.8)			
TRACT assessment: *High risk*	4	1950	Miso: 48/973 (4.9)	1.03 (0.86; 1.25)	Two‐stage	*I* ^2^ = 0.0%, *χ* ^2^ = 0.66, df = 3 (*p* = 0.883)
			Oxy: 44/977 (4.5)			
Trustworthy data (IPD+*low risk* RCTs)	20	25 249	Miso: 480/12 746 (3.8)	1.36 (1.16; 1.59)	Two‐stage	—
			Oxy: 355/12 503 (2.8)			
Data not meeting trustworthy criteria (*moderate+high risk* RCTs)	14	5515	Miso: 61/2823 (2.2)	1.02 (0.85; 1.24)	Two‐stage	—
			Oxy: 58/2692 (2.2)			
All data: IPD+aggregate data (*low/moderate/high risk* RCTs)	34	30 764	Miso: 541/15 569 (3.5)	1.19 (1.02; 1.40)	Two‐stage	*χ* ^2^ = 8.97, df = 3 (*p* = 0.030)
			Oxy: 413/15 195 (2.7)			

Abbreviations: aOR: adjusted odds ratio; CI: confidence interval; IPD: individual participant data; MD: mean difference; Miso: misoprostol; Oxy: oxytocin; PPH: postpartum haemorrhage; TRACT: Trustworthiness in RAndomised Controlled Trials; WHO: World Health Organisation.

^a^ One‐stage meta‐analysis adjusted for maternal age, parity and gestational age.

** *p*‐value for significance.

Regarding PPH ≥ 1000 mL, analysis of trustworthy data showed that misoprostol was associated with a significantly increased risk (20 RCTs, 25 249 participants, OR 1.36, 95% CI 1.16–1.59, moderate certainty evidence, Tables 2 and S8, Figure 3). Analysis of data not meeting our trustworthiness criteria showed misoprostol has a similar efficacy to oxytocin for PPH ≥ 1000 mL (14 RCTs, 5515 participants, OR 1.02, 95% CI 0.85–1.24).

#### 4.6.6 Integration of IPD and Aggregate Data MA for Secondary Outcomes

Due to significant clinical heterogeneity, secondary outcomes could be assessed with aggregate data.

#### 4.6.7 Integration of IPD and Aggregate Data MA for Subgroup Analysis

We performed a subgroup analysis according to the route of misoprostol delivery, PO (oral) and SL, and separately, PR. Analysis of PO/SL misoprostol IPD indicated that misoprostol is associated with a significant increase in PPH ≥ 500 mL (5 RCTs, 1742 participants, OR 1.85, 95% CI 1.30–2.63; Figure S3). Analysis of trustworthy data showed that misoprostol is associated with a non‐significant increase in PPH ≥ 500 mL (13 RCTs, 11 554 participants, OR 1.25, 95% CI 0.85–1.84). Analysis of PR misoprostol IPD showed that misoprostol is associated with a non‐significant increase in PPH ≥ 500 mL (2 RCTs, 280 participants, OR 1.60, 95% CI 0.03–84.62; Figure S4). Analysis of trustworthy data shows that misoprostol is associated with a non‐significant decrease in PPH ≥ 500 mL (5 RCTs, 1226 participants, OR 0.90, 95% CI 0.45–1.83).

Analysis of PO/SL misoprostol IPD shows that misoprostol is associated with a non‐significant increase in PPH ≥ 1000 mL (4 RCTs, 1742 participants, OR 1.14, 95% CI 0.64–2.04; Figure S5). Analysis of trustworthy data shows that misoprostol is associated with a significant increase in PPH ≥ 1000 mL (15 RCTs, 22 281 participants, OR 1.38, 95% CI 1.21–1.58). We were unable to analyse PR misoprostol IPD as only two RCTs used PR misoprostol, and both studies reported zero events. Analysis of trustworthy data showed that PR misoprostol was associated with a non‐significant decrease in PPH ≥ 1000 mL (5 RCTs, 1226 participants, OR 0.55, 95% CI 0.04–7.99; Figure S6).

We performed a subgroup analysis according to the method of blood loss measurement: estimated and measured. Regarding RCTs that used visual estimation of blood loss, analysis of trustworthy data showed that misoprostol was associated with a non‐significant increase in PPH ≥ 500 mL (6 RCTs, 1888 participants, OR 1.12, 95% CI 0.38–3.31; Figure S7), and PPH ≥ 1000 mL (7 RCTs, 2419 participants, OR 1.57, 95% CI 0.78–3.15; Figure S9) as compared to oxytocin.

IPD from RCTs that measured blood loss suggested that misoprostol is associated with a significantly increased risk of PPH ≥ 500 mL (5 RCTs, 1720 participants, OR 1.81, 95% CI 1.38–2.38; Figure S8); trustworthy data show that misoprostol is associated with a non‐significant increased risk of PPH ≥ 500 mL (12 RCTs, 22 446 participants, OR 1.15, 95% CI 0.80–1.65; Figure S8) as compared to oxytocin. IPD‐MA of RCTs that measured blood loss showed that misoprostol was associated with a non‐significant increase in PPH ≥ 1000 mL (5 RCTs, 1720 participants, OR 1.16, 95% CI 0.10–13.63; Figure S10). Analysis of trustworthy data similarly showed that misoprostol was associated with a non‐significant increased risk of PPH ≥ 1000 mL (12 RCTs, 22 448 participants, OR 1.25, 95% CI 0.91–1.71; Figure S10).

Subgroup analysis was unable to be performed according to MOD due to a lack of data availability. Of 43 RCTs reporting PPH ≥ 500 mL, only 4 were in a CS delivery population. Therefore, we were unable to perform a subgroup aggregate data MA according to data trustworthiness comparing CS to vaginal delivery populations. A post hoc subgroup analysis for vaginal MOD was performed with both IPD and aggregate data; trustworthy data showed that misoprostol has similar efficacy to oxytocin for preventing PPH ≥ 500 mL (14 RCTs, 23 904 participants, OR 1.03, 95% CI 0.62–1.71; Figure S11) and that misoprostol is associated with a significant increase in PPH ≥ 1000 mL (15 RCTs, 24 463 participants, OR 1.41, 95% CI 1.26–1.56; Figure S12).

#### 4.6.8 Sensitivity Analysis

An ‘as‐treated’ sensitivity analysis was unable to be performed with IPD, as all participants received their allocated treatment. One IPD trial publication specifically described that there was no misallocation of treatment [22]. Other IPD did not provide information on misallocation of participants.

## 5 Discussion

### 5.1 Main Findings

Jointly considering the results of IPD‐MA and aggregate MA of all RCTs meeting trustworthiness criteria, we found that oxytocin is at least comparable to misoprostol in terms of PPH prevention and may be superior for preventing severe PPH. Challenges in assuring the certainty of evidence lie in the fact that many of the RCTs comparing misoprostol and oxytocin did not meet trustworthiness criteria. Also, a few RCTs provided IPD, making it not possible to fully explore the heterogeneity between RCTs.

In comparison to the 2018 Cochrane NMA, our analysis included an IPD‐MA in addition to the aggregate data MA. Our updated search included a further 17 RCTs. Of these RCTs, we received IPD from three, one of which was assessed as trustworthy and was used in our IPD‐MA. Analysis of our trustworthy data confirmed the Cochrane NMA findings that oxytocin and misoprostol are not significantly different for the prevention of PPH ≥ 500 mL, and that oxytocin is associated with a significant reduction in PPH ≥ 1000 mL as compared to misoprostol.

### 5.2 Strengths and Limitations

The main strength of this study is the IPD‐MA design, which allows data trustworthiness to be verified prior to analysis, enables outcome harmonisation, and treatment‐covariate interaction analysis. The sample size of 2022 participants, while only coming from 6 of 79 eligible RCTs, is large. The largest study contributed just over 50% of the total data [22], the findings are unlikely to be driven by one study. The central research team continuously communicated with trial investigators to ensure data consistency and quality.

Thirty‐one of 79 RCTs (39%) did not respond to our IPD‐MA invitations, and a further 38 RCTs (48%) declined to participate. Concerningly, many non‐responding RCTs were published recently. While we potentially lost more than half of the available evidence, we may have filtered out non‐trustworthy data. If all 79 trials contributed data, there would have been 44 941 participants. IPD from only 10 trials was received, but after data checking, only 6 of these were included in the MA. This presents a risk of data availability bias. However, data availability bias assumes that all data are of high quality and are trustworthy.

Many IPD variables were recorded differently by trialists. During data harmonisation, some variables had to conform to binary categorisation, which may have resulted in a loss of information. For example, the largest study (1140 participants) [22] categorised participant age as 18–35 and > 35 years; therefore, the remaining IPD conformed to this categorisation. Likewise, six datasets were required to conform to the binary categorisation of nulliparous or multiparous.

Similarly, regarding the aggregate data, the outcomes were derived and presented differently across all RCTs; therefore, many outcomes were unable to be assessed in the MA. For example, most RCTs reported PPH ≥ 500 or ≥ 1000 mL, plus a further measure of blood loss, either EBL, Hb drop, HCT drop, additional uterotonic use or blood transfusion requirement. Within each outcome, the results were presented differently; some RCTs presented EBL as mean ± standard deviation (SD) or mean and CI, median and quartiles, or just mean alone. After RCT stratification into low‐, moderate‐ or high‐risk for trustworthiness concerns, there were not enough data to perform MA for EBL, Hb drop, HCT drop, additional uterotonic use or blood transfusion requirement outcomes, given the inconsistent presentations of these results.

The main limitation of this meta‐analysis was the clinical heterogeneity of the included trials. Trial heterogeneity of the low‐ and moderate‐risk RCTs was greatest. We were unable to understand the difference in PPH prevalence across trials. In a vaginal delivery population, the prevalence of PPH ≥ 500 mL ranged between 0% [35, 36, 37, 38] and 100% [36]. In studies that were low risk for data integrity concerns, the prevalence of PPH ≥ 500 mL ranged from 0% to 45.5% [39, 40, 41]; IPD ranged from 0% to 28.5% [22, 26]. The variation in PPH prevalence may be due to trial heterogeneity with respect to the year, country, economic status (high‐, middle‐ or low‐income countries), sample size, dose and route of study medications, or due to the inherently subjective nature of measuring postpartum blood loss, which is a well‐described problem [42]. Because the varied PPH prevalence of these trials is not understood, there may be limited benefit in meta‐analysis and attempting to generalise results.

Finally, our trustworthiness assessment presents both a strength and a potential limitation. Given the increasing evidence of problematic data permeating women's health, it is imperative to assess data trustworthiness prior to integration in evidence synthesis [43]. Without a trustworthiness assessment, there is a risk that problematic data are integrated into MA and can subsequently impact clinical guidelines and patient care. For example, regarding the management of recurrent miscarriage with progesterone and the management of impacted fetal head in caesarean section with the fetal pillow, the clinical recommendations for these conditions changed following the removal of untrustworthy data from evidence synthesis [44, 45]. However, the application of data integrity tools such as the TRACT checklist [21] and other similar checklists [46, 47] might have an arbitrary and partly subjective character. These tools are relatively new, and more experience with screening for trustworthiness issues will improve standardisation. However, the fact that many authors did not even respond to our request for data is concerning. Also, many papers had co‐authors with retracted papers or had clear signs of research misconduct, which is an indication that this issue can no longer remain unaddressed [48, 49, 50, 51]. It is irrelevant whether data are intentionally fabricated or whether they do not meet trustworthiness standards; data that are not trustworthy should not be included in meta‐analyses that inform clinical practice [52].

### 5.3 Interpretation

Current literature supports the use of active management in the third stage of labour with a uterotonic for the prevention of PPH. Most obstetric guidelines recommend 10 IU of oxytocin [11, 12, 53, 54]. Many of these guidelines [10, 11, 54] are based on the 2018 Cochrane NMA, which preferred oxytocin for routine prevention of PPH for its relative lack of maternal side effects [5]. In 2023, the National Institute for Healthcare Excellence (NICE) found that misoprostol is more effective at preventing PPH than oxytocin, but did not recommend misoprostol in their guideline due to its maternal side effects [55].

The Cochrane NMA found that misoprostol and oxytocin were similarly effective for PPH ≥ 500 mL and that misoprostol was associated with a significantly increased risk of PPH ≥ 1000 mL. In contrast, our IPD‐MA included 2022 participants and found that misoprostol was associated with a significantly increased risk of PPH ≥ 500 mL and a non‐significant increased risk of severe PPH ≥ 1000 mL as compared to oxytocin. Our analysis of trustworthy data included over 22 000 participants and found that misoprostol had similar efficacy to oxytocin for PPH ≥ 500 mL and that misoprostol was associated with a significantly increased risk of PPH ≥ 1000 mL as compared to oxytocin. The similarity in our trustworthy analysis and the Cochrane NMA is understandable as a large WHO trial with almost 20 000 participants received a large weight in both analyses and showed a clear benefit of oxytocin over misoprostol. Furthermore, trials that were omitted from our trustworthy analysis frequently reported no bleeding events in one or both treatment arms and therefore were excluded from Cochrane's meta‐analysis as their treatment effect was not estimable.

In this IPD‐MA, there were 79 RCTs comparing misoprostol and oxytocin. The large number of trials may be explained in part by dose‐finding trials or studies focusing on specific patient groups. However, many of these RCTs were conducted recently and were not registered. One may question whether the methodology of these RCTs was robust and if their results are trustworthy. Identifying and managing RCTs with data integrity concerns is a new and often controversial area of research. However, the need to screen publications for trustworthiness concerns or to retrieve IPD is obvious. IPD allows data trustworthiness to be tested, ensuring that trials with compromised data integrity are omitted from meta‐analysis and subsequent guideline development.

The findings of our IPD‐MA demonstrate that oxytocin is at least comparable to misoprostol for preventing PPH and may be superior for preventing severe PPH. These findings support current WHO recommendations for the routine use of oxytocin to prevent PPH [10]. Unfortunately, there are barriers to oxytocin administration that can make widespread use in lower‐income countries difficult. Oxytocin is temperature sensitive and requires transport and refrigeration between 2°C and 8°C, and IM or IV administration by trained healthcare workers. In recognition of these limitations and because misoprostol is heat stable and easy to administer, in 2015, the WHO recommended oral misoprostol for the prevention and treatment of PPH as a valid alternative [10, 56]. For women living in poverty, among displaced populations, in conflict areas or rural areas, access to refrigeration and skilled healthcare workers may not be possible, and thus, oral misoprostol presents a feasible alternative in low‐resource settings [57]. Our findings support the use of oral misoprostol as an equivalent agent to oxytocin for preventing PPH; however, where possible, oxytocin should be used for preventing severe PPH.

### 5.4 Conclusions and Implications

Less than half (37%) of the RCTs comparing misoprostol and oxytocin for the prevention of PPH meet trustworthiness criteria based on raw data or assessment of the published paper. Routine screening for the trustworthiness of RCTs will assist in building the robustness of evidence synthesis. Oxytocin is at least comparable to misoprostol for preventing PPH and may be superior for preventing severe PPH.

## Author Contributions

M.F. principally managed the project and the collaborative process and carried out design, data collection, IPD checking, data synthesis, writing, editing, finalising, and submitting of the manuscript. B.W.M., W.L., and D.L.R. conceived the research idea and were responsible for overseeing all aspects of the conduct. A.R. and M.P. contributed to eligibility screening and risk of bias assessment as the second and third reviewers. D.L.R. and B.W.M. provided clinical oversight. W.L. and N.A. provided statistical oversight. M.F. designed and conducted the literature searches. R.S., D.S., E.T., V.J., A.O.M. and M.A.I. prepared and supplied their data, answered questions regarding their data and contributed to editing and finalising the manuscript. All authors were involved in the decision to submit the manuscript. All contributing trial investigators had opportunities to comment on the initial scope, draft protocol, and draft statistical analysis plan and participated in teleconference meetings as the project progressed.

## Disclosure

This study is supported by two NHMRC Investigator Grants (GNT1176437 for B.W.M. and GNT117647 for W.L.). These funding sources had no role in the design, execution, analyses, or data interpretation for this research. M.P. and M.F. are supported by a Research Training Stipend, provided by the Australian Government. B.W.M. reports consultancy, travel support and research funding from Merck and consultancy for Organon and Norgine. B.W.M. holds stock from ObsEva.

## Ethics Statement

Ethics approval was obtained from the Monash University Human Research Ethics Committee on 13/7/2022 (Project ID#34839).

## Conflicts of Interest

The authors declare no conflicts of interest.

## Supporting information


**Table S1:** Definition of outcomes and potential effect modifiers.
**Table S2:** Overview of all studies included in the MA comparing misoprostol versus oxytocin for the prevention of PPH (outcomes of contact with authors and TRACT assessment).
**Table S3:** Outline of IPD excluded from MA: concerns and trialist response to concerns.
**Table S4:** Baseline characteristics of IPD.
**Table S5:** IPD‐MA: misoprostol compared to oxytocin for primary and secondary outcomes.
**Table S6:** Subgroup analysis according to BMI (kg/m^2^) and pre‐partum Hb (g/dL): misoprostol compared with oxytocin for the outcome PPH ≥ 500 and ≥ 1000 mL.
**Table S7:** Intervention covariate interactions for primary outcomes.
**Table S8:** GRADE assessment for primary outcomes according to the type of data used in meta‐analysis.
**Figure S1:** Risk of bias 2 (RoB‐2) assessment of included trials in the intention‐to‐treat population.
**Figure S2:** RoB 2 scoring domains.
**Figure S3:** Misoprostol compared to oxytocin for the outcome PPH ≥ 500 mL according to per os (oral) or sublingual misoprostol route of administration; forest plot comparison of IPD MA and aggregate data MA of RCTs, *low, moderate and high risk* for data integrity concerns (according to TRACT assessment).
**Figure S4:** Misoprostol compared to oxytocin for the outcome PPH ≥ 500 mL according to per rectal misoprostol route of administration; forest plot comparison of IPD MA and aggregate data MA of RCTs, *low, moderate and high risk* for data integrity concerns (according to TRACT assessment).
**Figure S5:** Misoprostol compared to oxytocin for the outcome PPH ≥ 1000 mL according to per os (oral) or sublingual misoprostol route of administration; forest plot comparison of IPD MA and aggregate data MA of RCTs, *low, moderate and high risk* for data integrity concerns (according to TRACT assessment).
**Figure S6:** Misoprostol compared to oxytocin for the outcome PPH ≥ 1000 mL according to per rectal misoprostol route of administration; forest plot comparison of IPD MA and aggregate data MA of RCTs, *low, moderate and high risk* for data integrity concerns (according to TRACT assessment).
**Figure S7:** Misoprostol compared to oxytocin for the outcome PPH ≥ 500 mL according to estimated (not measured) blood loss; forest plot comparison of IPD MA and aggregate data MA of RCTs, *low, moderate and high risk* for data integrity concerns (according to TRACT assessment).
**Figure S8:** Misoprostol compared to oxytocin for the outcome PPH ≥ 500 mL according to measured blood loss; forest plot comparison of IPD MA and aggregate data MA of RCTs, *low, moderate and high risk* for data integrity concerns (according to TRACT assessment).
**Figure S9:** Misoprostol compared to oxytocin for the outcome PPH ≥ 1000 mL according to estimated (not measured) blood loss; forest plot comparison of IPD MA and aggregate data MA of RCTs, *low, moderate and high risk* for data integrity concerns (according to TRACT assessment).
**Figure S10:** Misoprostol compared to oxytocin for the outcome PPH ≥ 1000 mL according to measured blood loss; forest plot comparison of IPD MA and aggregate data MA of RCTs, *low, moderate and high risk* for data integrity concerns (according to TRACT assessment).
**Figure S11:** Misoprostol compared to oxytocin for the outcome PPH ≥ 500 mL in a vaginal delivery population; forest plot comparison of IPD MA and aggregate data MA of RCTs *low, moderate and high risk* for data integrity concerns (according to TRACT assessment).
**Figure S12:** Misoprostol compared to oxytocin for the outcome PPH ≥ 1000 mL in a vaginal delivery population; forest plot comparison of IPD MA and aggregate data MA of RCTs *low, moderate and high risk* for data integrity concerns (according to TRACT assessment).

## Data Availability

The protocol, statistical analysis plan, and codebook are available on request. The trial investigators who shared individual participant data for the purposes of the meta‐analysis retain ownership of their trial data, and any requests for access to individual participant data should be made directly to them.
