# Supplementary material for: Misoprostol Versus Oxytocin for the Prevention of Postpartum Haemorrhage: A Systematic Review and Meta‐Analysis Including Individual Participant Data
Source: BJOG. 2025 May 13;132(10):1364–77. doi: 10.1111/1471-0528.18197 (PMC12315072; doi:10.1111/1471-0528.18197)
Supplement: Supplementary file 1 — Table S1: Definition of outcomes and potential effect modifiers. Table S2: Overview of all studies included in the MA comparing misoprostol versus oxytocin for the prevention of PPH (outcomes of contact with authors and TRACT assessment). Table S3: Outline of IPD excluded from MA: concerns and trialist response to concerns. Table S4: Baseline characteristics of IPD. Table S5: IPD‐MA: misoprostol compared to oxytocin for primary and secondary outcomes. Table S6: Subgroup analysis according to BMI (kg/m2) and pre‐partum Hb (g/dL): misoprostol compared with oxytocin for the outcome PPH ≥ 500 and ≥ 1000 mL. Table S7: Intervention covariate interactions for primary outcomes. Table S8: GRADE assessment for primary outcomes according to the type of data used in meta‐analysis. Figure S1: Risk of bias 2 (RoB‐2) assessment of included trials in the intention‐to‐treat population. Figure S2: RoB 2 scoring domains. Figure S3: Misoprostol compared to oxytocin for the outcome PPH ≥ 500 mL according to per os (oral) or sublingual misoprostol route of administration; forest plot comparison of IPD MA and aggregate data MA of RCTs, low, moderate and high risk for data integrity concerns (according to TRACT assessment). Figure S4: Misoprostol compared to oxytocin for the outcome PPH ≥ 500 mL according to per rectal misoprostol route of administration; forest plot comparison of IPD MA and aggregate data MA of RCTs, low, moderate and high risk for data integrity concerns (according to TRACT assessment). Figure S5: Misoprostol compared to oxytocin for the outcome PPH ≥ 1000 mL according to per os (oral) or sublingual misoprostol route of administration; forest plot comparison of IPD MA and aggregate data MA of RCTs, low, moderate and high risk for data integrity concerns (according to TRACT assessment). Figure S6: Misoprostol compared to oxytocin for the outcome PPH ≥ 1000 mL according to per rectal misoprostol route of administration; forest plot comparison of IPD MA and aggregate data M [file BJO-132-1364-s001.docx]

**Summary of Appendices:**

**Appendix A:** PRISMA 2020 checklist**.**

**Apendix B:** PRISMA 2020 abstract checklist**.**

**Appendix C:** Search strategy used to identify RCTs comparing misoprostol versus oxytocin for the prevention of PPH in Ovid databases.

**Appendix D:** Search strategy used to identify RCTs comparing misoprostol versus oxytocin for the prevention of PPH in EBSCO CINAHL database.

**Appendix E:** Search strategy used to identify RCTs comparing misoprostol versus oxytocin for the prevention of PPH in *clinicaltrials.gov* database.

**Appendix F:** Search strategy used to identify RCTs comparing misoprostol versus oxytocin for the prevention of PPH in *Scopus* database.

**Appendix G:** The Trustworthiness in RAndomised Controlled Trials (TRACT) tool

**Appendix H:** List of included RCTs for which enquiry was sent, and method and description of blood loss measurement.

**Appendix I:** List of RCTs in full text screening that were excluded.

**Appendix J:** Funnel plots for the primary outcome PPH≥500mL.

**Appendix K:** Funnel plots for the primary outcome PPH≥1000mL.

**Appendix A:** PRISMA 2020 Checklist

| **Section and Topic** | **Item #** | **Checklist item** | **Location where item is reported** |
| --- | --- | --- | --- |
| **TITLE** | | |  |
| Title | 1 | Identify the report as a systematic review. | Individual participant data meta-analysis- specified in title, Page 1 |
| **ABSTRACT** | | |  |
| Abstract | 2 | See the PRISMA 2020 for Abstracts checklist. | See below, Appendix B. |
| **INTRODUCTION** | | |  |
| Rationale | 3 | Describe the rationale for the review in the context of existing knowledge. | Page 4, line 23 |
| Objectives | 4 | Provide an explicit statement of the objective(s) or question(s) the review addresses. | Page 4, line 30 |
| **METHODS** | | |  |
| Eligibility criteria | 5 | Specify the inclusion and exclusion criteria for the review and how studies were grouped for the syntheses. | Page 5, line 42 |
| Information sources | 6 | Specify all databases, registers, websites, organisations, reference lists and other sources searched or consulted to identify studies. Specify the date when each source was last searched or consulted. | Page 5, line 48 |
| Search strategy | 7 | Present the full search strategies for all databases, registers and websites, including any filters and limits used. | Appendix C- F |
| Selection process | 8 | Specify the methods used to decide whether a study met the inclusion criteria of the review, including how many reviewers screened each record and each report retrieved, whether they worked independently, and if applicable, details of automation tools used in the process. | Page 5, line 52 |
| Data collection process | 9 | Specify the methods used to collect data from reports, including how many reviewers collected data from each report, whether they worked independently, any processes for obtaining or confirming data from study investigators, and if applicable, details of automation tools used in the process. | Page 5, line 54 |
| Data items | 10a | List and define all outcomes for which data were sought. Specify whether all results that were compatible with each outcome domain in each study were sought (e.g. for all measures, time points, analyses), and if not, the methods used to decide which results to collect. | Page 6, line 71  Supplementary Table 1 |
|  | 10b | List and define all other variables for which data were sought (e.g. participant and intervention characteristics, funding sources). Describe any assumptions made about any missing or unclear information. | Page 6, line 90  Supplementary Table 1 & 6  Statistical analysis plan (SAP), available from: <https://www.crd.york.ac.uk/prospero/>); CRD42022343340. |
| Study risk of bias assessment | 11 | Specify the methods used to assess risk of bias in the included studies, including details of the tool(s) used, how many reviewers assessed each study and whether they worked independently, and if applicable, details of automation tools used in the process. | Page 6, line 67 |
| Effect measures | 12 | Specify for each outcome the effect measure(s) (e.g. risk ratio, mean difference) used in the synthesis or presentation of results. | Page 7, line 101 |
| Synthesis methods | 13a | Describe the processes used to decide which studies were eligible for each synthesis (e.g. tabulating the study intervention characteristics and comparing against the planned groups for each synthesis (item #5)). | Page 7, line 108 |
|  | 13b | Describe any methods required to prepare the data for presentation or synthesis, such as handling of missing summary statistics, or data conversions. | Page 7, line 103 |
|  | 13c | Describe any methods used to tabulate or visually display results of individual studies and syntheses. | Page 7, line 110 |
|  | 13d | Describe any methods used to synthesize results and provide a rationale for the choice(s). If meta-analysis was performed, describe the model(s), method(s) to identify the presence and extent of statistical heterogeneity, and software package(s) used. | Page 6, line 94  Page 7, line 116 |
|  | 13e | Describe any methods used to explore possible causes of heterogeneity among study results (e.g. subgroup analysis, meta-regression). | Page 6, line 74 |
|  | 13f | Describe any sensitivity analyses conducted to assess robustness of the synthesized results. | Page 6, line 75 |
| Reporting bias assessment | 14 | Describe any methods used to assess risk of bias due to missing results in a synthesis (arising from reporting biases). | Page 6, line 75 |
| Certainty assessment | 15 | Describe any methods used to assess certainty (or confidence) in the body of evidence for an outcome. | Page 6, line 83 |
| **RESULTS** | | |  |
| Study selection | 16a | Describe the results of the search and selection process, from the number of records identified in the search to the number of studies included in the review, ideally using a flow diagram. | Page 7, line 122  Figure 1 |
|  | 16b | Cite studies that might appear to meet the inclusion criteria, but which were excluded, and explain why they were excluded. | Appendix H |
| Study characteristics | 17 | Cite each included study and present its characteristics. | Supplementary Table 2, Appendix G |
| Risk of bias in studies | 18 | Present assessments of risk of bias for each included study. | Supplementary Figure 1 and 2 |
| Results of individual studies | 19 | For all outcomes, present, for each study: (a) summary statistics for each group (where appropriate) and (b) an effect estimate and its precision (e.g. confidence/credible interval), ideally using structured tables or plots. | Figure 3, Figure 4,  Supplementary Table 5 |
| Results of syntheses | 20a | For each synthesis, briefly summarise the characteristics and risk of bias among contributing studies. | Page 8, line 132  Page 8, Line 138  Page 8, line 145 |
|  | 20b | Present results of all statistical syntheses conducted. If meta-analysis was done, present for each the summary estimate and its precision (e.g. confidence/credible interval) and measures of statistical heterogeneity. If comparing groups, describe the direction of the effect. | Figure 3, Figure 4  Supplementary Table 8 & 9 |
|  | 20c | Present results of all investigations of possible causes of heterogeneity among study results. | Page 9, line  Supplementary Figure 3 & 4 |
|  | 20d | Present results of all sensitivity analyses conducted to assess the robustness of the synthesized results. | Supplementary Figure 3 & 4 |
| Reporting biases | 21 | Present assessments of risk of bias due to missing results (arising from reporting biases) for each synthesis assessed. |  |
| Certainty of evidence | 22 | Present assessments of certainty (or confidence) in the body of evidence for each outcome assessed. | Appendix I |
| **DISCUSSION** | | |  |
| Discussion | 23a | Provide a general interpretation of the results in the context of other evidence. | Page 10, line 208 |
|  | 23b | Discuss any limitations of the evidence included in the review. | Page 10, line 223 |
|  | 23c | Discuss any limitations of the review processes used. | Page 11, line 246 |
|  | 23d | Discuss implications of the results for practice, policy, and future research. | Page 11, line 254 |
| **OTHER INFORMATION** | | |  |
| Registration and protocol | 24a | Provide registration information for the review, including register name and registration number, or state that the review was not registered. | Page 5, line 37 |
|  | 24b | Indicate where the review protocol can be accessed, or state that a protocol was not prepared. | Page 5, line 38 |
|  | 24c | Describe and explain any amendments to information provided at registration or in the protocol. | - |
| Support | 25 | Describe sources of financial or non-financial support for the review, and the role of the funders or sponsors in the review. | Page 3. |
| Competing interests | 26 | Declare any competing interests of review authors. | Page 14, line 295 |
| Availability of data, code and other materials | 27 | Report which of the following are publicly available and where they can be found: template data collection forms; data extracted from included studies; data used for all analyses; analytic code; any other materials used in the review. | Page 14, line 316 (statement on data sharing) |

| **Section and Topic** | **Item #** | **Checklist item** | **Reported (Yes/No)** |
| --- | --- | --- | --- |
| **TITLE** | | |  |
| Title | 1 | Identify the report as a systematic review. | Individual participant data meta-analysis- specified in title, Page 1 |
| **BACKGROUND** | | |  |
| Objectives | 2 | Provide an explicit statement of the main objective(s) or question(s) the review addresses. | Yes |
| **METHODS** | | |  |
| Eligibility criteria | 3 | Specify the inclusion and exclusion criteria for the review. | Yes |
| Information sources | 4 | Specify the information sources (e.g. databases, registers) used to identify studies and the date when each was last searched. | (No, in body of manuscript) |
| Risk of bias | 5 | Specify the methods used to assess risk of bias in the included studies. | (No, in body of manuscript) |
| Synthesis of results | 6 | Specify the methods used to present and synthesise results. | Yes |
| **RESULTS** | | |  |
| Included studies | 7 | Give the total number of included studies and participants and summarise relevant characteristics of studies. | Yes |
| Synthesis of results | 8 | Present results for main outcomes, preferably indicating the number of included studies and participants for each. If meta-analysis was done, report the summary estimate and confidence/credible interval. If comparing groups, indicate the direction of the effect (i.e. which group is favoured). | Yes |
| **DISCUSSION** | | |  |
| Limitations of evidence | 9 | Provide a brief summary of the limitations of the evidence included in the review (e.g. study risk of bias, inconsistency and imprecision). | Yes |
| Interpretation | 10 | Provide a general interpretation of the results and important implications. | Yes |
| **OTHER** | | |  |
| Funding | 11 | Specify the primary source of funding for the review. | Yes |
| Registration | 12 | Provide the register name and registration number. | (No, in body of manuscript) |

**Appendix B:** PRISMA 2020 Abstract Checklist

**Appendix C:** Search strategy used to identify RCTs comparing misoprostol versus oxytocin for the prevention of PPH. This search was last conducted in all Ovid databases (Medline, Embase, Emcare, Cochrane Central Register of Controlled Trials); October, 2024:

| EBM Reviews - Cochrane Central Register of Controlled Trials <September 2024>  EBM Reviews - Cochrane Database of Systematic Reviews <2005 to October 16, 2024>  Embase Classic+Embase <1947 to 2024 October 21>  Ovid Emcare <1995 to 2024 Week 41>  Ovid MEDLINE(R) and Epub Ahead of Print, In-Process, In-Data-Review & Other Non-Indexed Citations, Daily and Versions <1946 to October 21, 2024>  1 exp Delivery, Obstetric/ 398124  2 exp Labor, Obstetric/ 111623  3 exp Parturition/ 69255  4 (third stage or labo?r or childbirth* or delivery* of birth* or parturit* or parit*).mp. [mp=ti, ot, ab, fx, sh, hw, kw, tx, ct, tn, dm, mf, dv, kf, dq, bt, nm, ox, px, rx, ui, sy, ux, mx] 791647  5 exp Prostaglandins, Synthetic/ 27022  6 exp Oxytocics/ 81563  7 (carboprost or dinoprost or dinoprostone or ergonovine or ergotamine or methylergonovine or misoprostol or oxytocin or quizapine or sparteine or vasotocin).mp. [mp=ti, ot, ab, fx, sh, hw, kw, tx, ct, tn, dm, mf, dv, kf, dq, bt, nm, ox, px, rx, ui, sy, ux, mx] 187640  8 (active management or pitocin or syntocinon or prostoglandin*).mp. [mp=ti, ot, ab, fx, sh, hw, kw, tx, ct, tn, dm, mf, dv, kf, dq, bt, nm, ox, px, rx, ui, sy, ux, mx] 10192  9 postpartum haemorrhage.mp. [mp=ti, ot, ab, fx, sh, hw, kw, tx, ct, tn, dm, mf, dv, kf, dq, bt, nm, ox, px, rx, ui, sy, ux, mx] 9250  10 (((blood adj2loss and postpartum) or post partum or postnatal or post natal or obstetric) and (bleed* or h?em?or?hag*)).mp. [mp=ti, ot, ab, fx, sh, hw, kw, tx, ct, tn, dm, mf, dv, kf, dq, bt, nm, ox, px, rx, ui, sy, ux, mx] 54299  11 ((postpartum or post partum or postnatal or post natal or obstetric) and (bleed* or h?em?or?hag*)).mp. [mp=ti, ot, ab, fx, sh, hw, kw, tx, ct, tn, dm, mf, dv, kf, dq, bt, nm, ox, px, rx, ui, sy, ux, mx] 89527  12 9 or 10 or 11 89527  13 1 or 2 or 3 or 4 1066044  14 5 or 6 or 7 or 8 213940  15 12 and 13 and 14 9636  16 randomized controlled trials as topic/ 551741  17 randomized controlled trial/ 1758495  18 random allocation/ 247967  19 double blind method/ 622832  20 clinical trial/ 1919744  21 exp clinical trial as topic/ 489088  22 (clinic$ adj trial$1).tw. 1747938  23 ((singl$ or doubl$ or treb$ or tripl$) adj (blind$3 or mask$3)).tw. 942118  24 single blind method/ 136411  25 16 or 17 or 18 or 19 or 20 or 21 or 24 4173691  26 placebos/ 555651  27 placebo$.tw. 1139444  28 randomly allocated.tw. 159141  29 (allocated adj2 random).tw. 3498  30 22 or 23 or 26 or 27 or 28 or 29 3259090  31 25 or 30 5910743  32 case report.tw. 1191712  33 letter/ 2866129  34 historical article/ 372334  35 review of reported cases.pt. 0  36 Review, multicase.pt. 0  37 32 or 33 or 34 or 35 or 36 4395025  38 31 not 37 5790953  39 15 and 38 2993  40 39 2993  41 limit 40 to yr="2018 -Current" 1092  42 remove duplicates from 41 627 |
| --- |

**Appendix D (I-II):** Search strategy used to identify RCTs comparing misoprostol versus oxytocin for the prevention of PPH. I: This iteration of the search was conducted in EBSCO CINAHL, October, 2024 and was used for the PRISMA flowchart. II: This search was conducted to confirm that all relevant RCTs were captured by the initial search.

**D) I**

| 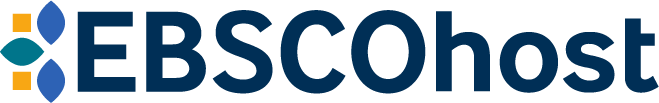 | Tue, October 22, 2024 03:43:44 pm |
| --- | --- |

| **#** | **Query** | **Limiters/Expanders** | **Last Run Via** | **Results** |
| --- | --- | --- | --- | --- |
| S28 | S26 AND S27 | Expanders - Apply equivalent subjects Search modes - Proximity | Interface - EBSCOhost Research Databases Search Screen - Advanced Search Database - CINAHL | 58 |
| S27 | S20 AND S23 AND S25 | Limiters - Publication Date: 20180101-20241031 Expanders - Apply equivalent subjects Search modes - Proximity | Interface - EBSCOhost Research Databases Search Screen - Advanced Search Database - CINAHL | 58 |
| S26 | S20 AND S23 AND S25 | Expanders - Apply equivalent subjects Search modes - Proximity | Interface - EBSCOhost Research Databases Search Screen - Advanced Search Database - CINAHL | 259 |
| S25 | S4 AND S11 | Expanders - Apply equivalent subjects Search modes - Proximity | Interface - EBSCOhost Research Databases Search Screen - Advanced Search Database - CINAHL | 5,150 |
| S24 | S14 AND S20 AND S23 | Expanders - Apply equivalent subjects Search modes - Proximity | Interface - EBSCOhost Research Databases Search Screen - Advanced Search Database - CINAHL | 0 |
| S23 | S21 OR S22 | Expanders - Apply equivalent subjects Search modes - Proximity | Interface - EBSCOhost Research Databases Search Screen - Advanced Search Database - CINAHL | 4,200 |
| S22 | (MH "Oxytocics+") | Expanders - Apply equivalent subjects Search modes - Proximity | Interface - EBSCOhost Research Databases Search Screen - Advanced Search Database - CINAHL | 4,200 |
| S21 | (MM "Oxytocin") | Expanders - Apply equivalent subjects Search modes - Proximity | Interface - EBSCOhost Research Databases Search Screen - Advanced Search Database - CINAHL | 1,475 |
| S20 | S17 OR S19 | Expanders - Apply equivalent subjects Search modes - Proximity | Interface - EBSCOhost Research Databases Search Screen - Advanced Search Database - CINAHL | 8,944 |
| S19 | S15 OR S18 | Expanders - Apply equivalent subjects Search modes - Proximity | Interface - EBSCOhost Research Databases Search Screen - Advanced Search Database - CINAHL | 3,311 |
| S18 | (MM "Prostaglandins, Synthetic+") | Expanders - Apply equivalent subjects Search modes - Proximity | Interface - EBSCOhost Research Databases Search Screen - Advanced Search Database - CINAHL | 2,065 |
| S17 | S15 OR S16 | Expanders - Apply equivalent subjects Search modes - Proximity | Interface - EBSCOhost Research Databases Search Screen - Advanced Search Database - CINAHL | 8,944 |
| S16 | (MH "Prostaglandins+") | Expanders - Apply equivalent subjects Search modes - Proximity | Interface - EBSCOhost Research Databases Search Screen - Advanced Search Database - CINAHL | 8,345 |
| S15 | (MM "Misoprostol") OR "misoprostol" | Expanders - Apply equivalent subjects Search modes - Proximity | Interface - EBSCOhost Research Databases Search Screen - Advanced Search Database - CINAHL | 2,713 |
| S14 | S12 AND S13 | Expanders - Apply equivalent subjects Search modes - Proximity | Interface - EBSCOhost Research Databases Search Screen - Advanced Search Database - CINAHL | 2 |
| S13 | (MH "Hemorrhage+") | Expanders - Apply equivalent subjects Search modes - Proximity | Interface - EBSCOhost Research Databases Search Screen - Advanced Search Database - CINAHL | 75,245 |
| S12 | S6 AND S11 | Expanders - Apply equivalent subjects Search modes - Proximity | Interface - EBSCOhost Research Databases Search Screen - Advanced Search Database - CINAHL | 2 |
| S11 | S7 OR S8 OR S9 OR S10 | Expanders - Apply equivalent subjects Search modes - Proximity | Interface - EBSCOhost Research Databases Search Screen - Advanced Search Database - CINAHL | 71,134 |
| S10 | (MM "Obstetric Emergencies") OR (MM "Delivery, Obstetric+") OR (MM "Obstetric Patients") OR (MM "Obstetric Service") OR (MM "Obstetric Care+") OR (MM "Pregnancy Outcomes") | Expanders - Apply equivalent subjects Search modes - Proximity | Interface - EBSCOhost Research Databases Search Screen - Advanced Search Database - CINAHL | 52,553 |
| S9 | (MM "Puerperium") OR (MM "Postpartum Hemorrhage") | Expanders - Apply equivalent subjects Search modes - Proximity | Interface - EBSCOhost Research Databases Search Screen - Advanced Search Database - CINAHL | 3,302 |
| S8 | (MH "Postnatal Period+") | Expanders - Apply equivalent subjects Search modes - Proximity | Interface - EBSCOhost Research Databases Search Screen - Advanced Search Database - CINAHL | 18,095 |
| S7 | (MM "Postpartum Hemorrhage") OR (MH "Postpartum Nursing") | Expanders - Apply equivalent subjects Search modes - Proximity | Interface - EBSCOhost Research Databases Search Screen - Advanced Search Database - CINAHL | 2,956 |
| S6 | (MM "Blood Loss Estimation") AND (MM "Blood Loss, Surgical") | Expanders - Apply equivalent subjects Search modes - Proximity | Interface - EBSCOhost Research Databases Search Screen - Advanced Search Database - CINAHL | 15 |
| S5 | (MM "Labor Complications+") OR (MM "Labor Stage, Third") AND N2 labo?r | Expanders - Apply equivalent subjects Search modes - Proximity | Interface - EBSCOhost Research Databases Search Screen - Advanced Search Database - CINAHL | 9,662 |
| S4 | S1 OR S2 OR S3 | Expanders - Apply equivalent subjects Search modes - Proximity | Interface - EBSCOhost Research Databases Search Screen - Advanced Search Database - CINAHL | 11,045 |
| S3 | (MM "Labor Stage, Third") | Expanders - Apply equivalent subjects Search modes - Proximity | Interface - EBSCOhost Research Databases Search Screen - Advanced Search Database - CINAHL | 365 |
| S2 | (MM "Labor Complications+") | Expanders - Apply equivalent subjects Search modes - Proximity | Interface - EBSCOhost Research Databases Search Screen - Advanced Search Database - CINAHL | 9,662 |
| S1 | (MH "Postpartum Hemorrhage") | Expanders - Apply equivalent subjects Search modes - Proximity | Interface - EBSCOhost Research Databases Search Screen - Advanced Search Database - CINAHL | 4,324 |

**D) II:**

| **#** | **Query** | **Limiters/Expanders** | **Last Run Via** | **Results** |
| --- | --- | --- | --- | --- |
| S30 | S19 AND S25 AND S29 | Limiters - Publication Date: 20180101-20250131; Randomized Controlled Trials Search modes - Proximity | Interface - EBSCOhost Research Databases Search Screen - Advanced Search Database - CINAHL Complete | 13 |
| S29 | S26 OR S27 OR S28 | Search modes - Proximity | Interface - EBSCOhost Research Databases Search Screen - Advanced Search Database - CINAHL Complete | 5,235 |
| S28 | "syntocinon or oxytocin" | Search modes - Proximity | Interface - EBSCOhost Research Databases Search Screen - Advanced Search Database - CINAHL Complete | 1 |
| S27 | oxytoc* | Search modes - Proximity | Interface - EBSCOhost Research Databases Search Screen - Advanced Search Database - CINAHL Complete | 5,235 |
| S26 | (MM "Oxytocin") | Search modes - Proximity | Interface - EBSCOhost Research Databases Search Screen - Advanced Search Database - CINAHL Complete | 1,545 |
| S25 | S22 OR S24 | Search modes - Proximity | Interface - EBSCOhost Research Databases Search Screen - Advanced Search Database - CINAHL Complete | 8,895 |
| S24 | S20 OR S23 | Search modes - Proximity | Interface - EBSCOhost Research Databases Search Screen - Advanced Search Database - CINAHL Complete | 3,301 |
| S23 | (MM "Prostaglandins, Synthetic+") | Search modes - Proximity | Interface - EBSCOhost Research Databases Search Screen - Advanced Search Database - CINAHL Complete | 2,059 |
| S22 | S20 OR S21 | Search modes - Proximity | Interface - EBSCOhost Research Databases Search Screen - Advanced Search Database - CINAHL Complete | 8,895 |
| S21 | (MH "Prostaglandins+") | Search modes - Proximity | Interface - EBSCOhost Research Databases Search Screen - Advanced Search Database - CINAHL Complete | 8,300 |
| S20 | (MM "Misoprostol") OR "misoprostol" | Search modes - Proximity | Interface - EBSCOhost Research Databases Search Screen - Advanced Search Database - CINAHL Complete | 2,705 |
| S19 | S4 OR S6 OR S14 OR S18 | Search modes - Proximity | Interface - EBSCOhost Research Databases Search Screen - Advanced Search Database - CINAHL Complete | 13,404 |
| S18 | S13 AND S17 | Search modes - Proximity | Interface - EBSCOhost Research Databases Search Screen - Advanced Search Database - CINAHL Complete | 1,847 |
| S17 | S15 OR S16 | Search modes - Proximity | Interface - EBSCOhost Research Databases Search Screen - Advanced Search Database - CINAHL Complete | 53,069 |
| S16 | h?em?orhag* | Search modes - Proximity | Interface - EBSCOhost Research Databases Search Screen - Advanced Search Database - CINAHL Complete | 4 |
| S15 | bleed* | Search modes - Proximity | Interface - EBSCOhost Research Databases Search Screen - Advanced Search Database - CINAHL Complete | 53,065 |
| S14 | S7 AND S13 | Search modes - Proximity | Interface - EBSCOhost Research Databases Search Screen - Advanced Search Database - CINAHL Complete | 1,480 |
| S13 | S8 OR S9 OR S10 OR S11 OR S12 | Search modes - Proximity | Interface - EBSCOhost Research Databases Search Screen - Advanced Search Database - CINAHL Complete | 107,087 |
| S12 | obstetric | Search modes - Proximity | Interface - EBSCOhost Research Databases Search Screen - Advanced Search Database - CINAHL Complete | 54,002 |
| S11 | post natal | Search modes - Proximity | Interface - EBSCOhost Research Databases Search Screen - Advanced Search Database - CINAHL Complete | 1,357 |
| S10 | postnatal | Search modes - Proximity | Interface - EBSCOhost Research Databases Search Screen - Advanced Search Database - CINAHL Complete | 37,449 |
| S9 | postpartum | Search modes - Proximity | Interface - EBSCOhost Research Databases Search Screen - Advanced Search Database - CINAHL Complete | 36,428 |
| S8 | postpartum | Search modes - Proximity | Interface - EBSCOhost Research Databases Search Screen - Advanced Search Database - CINAHL Complete | 36,428 |
| S7 | blood N2 loss | Search modes - Proximity | Interface - EBSCOhost Research Databases Search Screen - Advanced Search Database - CINAHL Complete | 17,562 |
| S6 | N2 labo?r | Search modes - SmartText Searching | Interface - EBSCOhost Research Databases Search Screen - Advanced Search Database - CINAHL Complete | 7 |
| S5 | S2 OR S3 | Search modes - Proximity | Interface - EBSCOhost Research Databases Search Screen - Advanced Search Database - CINAHL Complete | 10,072 |
| S4 | S1 OR S2 OR S3 | Search modes - Proximity | Interface - EBSCOhost Research Databases Search Screen - Advanced Search Database - CINAHL Complete | 11,249 |
| S3 | (MM "Labor Stage, Third") | Search modes - Proximity | Interface - EBSCOhost Research Databases Search Screen - Advanced Search Database - CINAHL Complete | 369 |
| S2 | (MM "Labor Complications+") | Search modes - Proximity | Interface - EBSCOhost Research Databases Search Screen - Advanced Search Database - CINAHL Complete | 9,843 |
| S1 | (MH "Postpartum Hemorrhage") | Search modes - Proximity | Interface - EBSCOhost Research Databases Search Screen - Advanced Search Database - CINAHL Complete | 4,366 |

**Appendix E:** Search strategy used to identify RCTs comparing misoprostol versus oxytocin for the prevention of PPH in *clinicaltrials.gov* database. This search was last conducted in October, 2024:

((PPH OR (postpartum haemorrhage OR postpartum hemorrhage OR post-partum hemorrhage OR post-partum haemorrhage OR postpartum bleeding) OR (haemorrhage OR hemorrhage OR bleeding) OR (postpartum OR puerperal OR post partum) AND (misoprostol OR oxytocin))


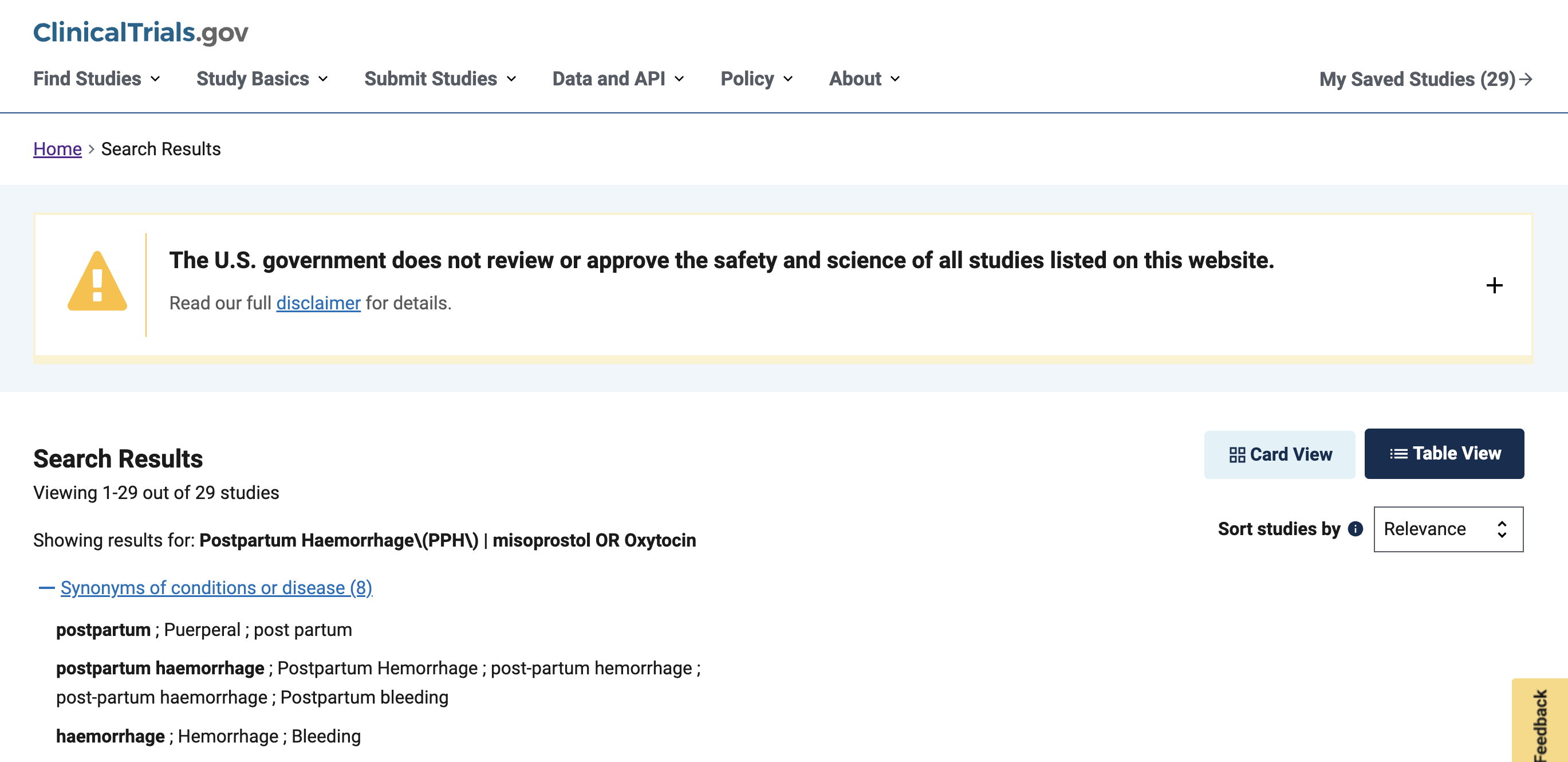


**Appendix F:** Search strategy used to identify RCTs comparing misoprostol versus oxytocin for the prevention of PPH in *Scopus* database. This search was last conducted in October, 2024:

( ( ( ALL ( ( active AND management OR pitocin OR syntocinon OR prostoglandin* ) ) ) OR ( ALL ( postpartum AND haemorrhage ) ) OR ( ALL ( ( ( ( blood AND adj2loss AND postpartum ) OR post AND partum OR postnatal OR post AND natal OR obstetric ) AND ( bleed* OR h?em?or?hag* ) ) ) ) OR ( ALL ( ( ( postpartum OR post AND partum OR postnatal OR post AND natal OR obstetric ) AND ( bleed* OR h?em?or?hag* ) ) ) ) ) AND ( ( ALL ( prostaglandin* ) ) OR ( ALL ( oxytocics ) ) OR ( ALL ( ( carboprost OR dinoprost OR dinoprostone OR ergonovine OR ergotamine OR methylergonovine OR misoprostol OR oxytocin OR quizapine OR sparteine OR vasotocin ) ) ) OR ( ALL ( ( active AND management OR pitocin OR syntocinon OR prostoglandin* ) ) ) ) AND ( ( TITLE-ABS-KEY ( delivery ) ) OR ( TITLE-ABS-KEY ( obstetric ) ) OR ( TITLE-ABS-KEY ( labor ) ) OR ( TITLE-ABS-KEY ( parturition ) ) OR ( TITLE-ABS-KEY ( ( third AND stage OR labo?r OR childbirth* OR delivery* OR parturit* OR parit* ) ) ) ) ) AND ( ( TITLE-ABS-KEY ( randomized AND controlled AND trials ) ) OR ( TITLE-ABS-KEY ( randomized AND controlled AND trial AND as AND topic ) ) OR ( TITLE-ABS-KEY ( random AND allocation ) ) OR ( TITLE-ABS-KEY ( double AND blind AND method ) ) OR ( TITLE-ABS-KEY ( clinical AND trial ) ) AND ( TITLE-ABS-KEY ( single AND blind AND method ) ) ) AND PUBYEAR > 2017 AND PUBYEAR < 2025

**Appendix G:** The Trustworthiness in RAndomised Controlled Trials (TRACT) tool

| 1.GOVERNANCE | |
| --- | --- |
| Y/N | Absent or retrospective registration of RCTs. This is relevant for RCTs commencing after 2010 |
| Y/N | Discrepancy in sample size in the RCT and trial registration |
| Y/N | Absent or vague description of research ethics or apparent concerns regarding ethics |
| 2. AUTHOR GROUP | |
| Y/N | Number of authors $\leq$3 or low author to study size ratio |
| Y/N | Other studies of authors have been retracted not on request of the authors   - Ran **all** authors through retraction watch database |
| Y/N | Large number of RCTs published in a small timeframe by one author/ in one institute |
| 3. PLAUSIBILITY OF INTERVENTION USAGE | |
| Y/N | Implausible use of placebo or intervention (e.g. two interventions but only one placebo) |
| Y/N | Use of sealed envelopes in a placebo-controlled trial |
| 4. TIMEFRAME | |
| Y/N | Fast recruitment of participants within the study time (especially single centre studies) |
| Y/N | Short or impossible time frame between ending recruitment/ follow up and submission of the paper (take into account time to outcome e.g. live birth, pregnancy outcome etc.) |
| 5. DROP-OUT RATES | |
| Y/N | Zero participants lost to follow up or no reasons mentioned for loss of follow up |
| Y/N | Ideal number of losses to follow up resulting in perfectly rounded number in each group (e.g. groups of 50 or 100) |
| 6. BASELINE CHARACTERISTICS | |
| Y/N | No or few baseline characteristics presented |
| Y/N | Implausible patient characteristics judging from common sense, the literature and local data (e.g. similar standard deviations for completely different characteristics with different means and distributions) |
| Y/N | Perfect balance for multiple baseline characteristics or significant/large differences between baseline characteristics |
| 7. OUTCOMES | |
| Y/N | Effect size that is much larger than in other RCTs regarding the same topic |
| Y/N | Conflicting information between outcomes (e.g. more ongoing pregnancies than clinical pregnancies) |

**Appendix H:** List of included RCTs for which enquiry was sent, and method and description of blood loss measurement (References to studies are listed in Supplementary Table 2).

|  | | | | **Blood loss measurement** | |
| --- | --- | --- | --- | --- | --- |
| **#** | **First Author** | **Year** | **Country** | **Method** | **Description** |
| **1** | Acharya^(2)^ | 2001 | UK | Visually estimated | Estimated by inspecting swabs/drapes/gauze/suction apparatus and pads, recorded until 1hr post op |
| **2** | Adanikin^(3)^ | 2012 | Nigeria | N/A | EBL not an outcome of this study |
| **3** | Adanikin^(4)^ | 2013 | Nigeria | Measured | Application of pads to the perineum of known weight, weighed by a weighing scale. |
| **4** | Afkham^(5)^ | 2022 | Iran | Not adequately stated | Gauze pad count was confirmed after the operation. |
| **5** | Afolabi^(6)^ | 2010 | Nigeria | Measured | Blood collected in a kidney dish, subsequently measured in a graduated measuring jar by the investigator. |
| **6** | Al-Sawaf^(7)^ | 2013 | Egypt | Measured | Pre weighed sterile packs. |
| **7** | Alwani^(8)^ | 2014 | India | N/A | EBL not an outcome of this study |
| **8** | Amin^(9)^ | 2014 | Pakistan | Measured | Blood collected in drapes that were weighed pre- and post-delivery. |
| **9** | Anita^(10)^ | 2018 | India | Measured | Measuring blood volume in suction bottle and blood-soaked sponges. |
| **10** | Asmat ^(11)^ | 2017 | Pakistan | Not stated | “Pads soaked were used to assess the amount of blood loss” |
| **11** | Atukunda^(12)^ | 2014 | Uganda | Measured | A clean plastic sheet specifically designed and piloted to collect blood for this trial was placed under the mother’s buttocks during and after the third stage of labor. Blood was drained into a calibrated container to improve accuracy in blood loss measurement [21,22]. All mothers were given pre-weighed standard sanitary pads to place in the perineum at all times. These pads were changed and weighed hourly for the first 6 h, and then every 6 h until 24 h postpartum. Blood loss was estimated as 1 ml per gram of weight of the pad after subtracting the dry pad weight, as previously described. |
| **12** | Baskett^(13)^ | 2007 | Canada | Visually estimated | Clinical estimate based on combination of visual assessment and measurement of blood volume in a kidney dish held under perineum during 3^rd^ stage. |
| **13** | Begum^(14)^ | 2015 | Bangladesh | Not stated | - |
| **14** | Bellad^(15)^ | 2012 | India | Measured | BRASSS-V drape transferred to measuring jar, blood soaked swabs, measured for 2hrs following delivery. |
| **15** | Benchimol^(16)^ | 2001 | France | Measured | Blood loss collection bag placed under patients buttocks and blood was weighed. |
| **16** | Bhatti^(17)^ | 2014 | Pakistan | Estimated | Visual assessment of blood loss, measured for 2 hours post delivery. |
| **17** | Bugalho^(18)^ | 2001 | Mozambique | Measured | Blood loss measured by placing a “metallic collector” under buttocks. |
| **18** | Burman^(19)^ | 2021 | India | Measured | Blood loss was measured objectively using the BRASS-VTM Drape, placed under the buttocks before delivery. The calibrated blood collection chamber was opened after delivery of the baby and drainage of amniotic fluid. The blood collected in the drape was measured in a separate jar with 10 mL calibrations. |
| **19** | Caliskan^(20)^ | 2002 | Turkey | Measured | Collected into a sterile steel bedpan after the birth by the help of a plastic bed linen, gauzes and pads were collected until 1hr after the delivery of the placenta. |
| **20** | Caliskan^(21)^ | 2003 | Turkey | Measured | Collected into a sterile steel bedpan after the birth by the help of a plastic bed linen, gauzes and pads were collected until 1hr after the delivery of the placenta. |
| **21** | Chaudhuri^(22)^ | 2010 | India | Measured | Estimated by adding volume of suction apparatus + soaked linen + mops. |
| **22** | Chaudhuri^(23)^ | 2012 | India | Measured | Specially designed, pre-weighed absorbent thick cotton pad with plastic lining was placed under the buttock of the woman. Blood clots, if any, were expressed from the vagina into a polythene bag. An episiotomy wound was repaired immediately, and the swabs used for that were not included in blood loss assess- ment. If necessary, the cotton pad under the buttock was replaced during the 1-hour observation period. One hour after delivery, the blood-soaked cotton pad(s) and the blood clots in the bag were weighed to assess blood loss. The specific gravity of blood being 1.08, the amount of blood lost in milliliters was approximately equal to the weight in grams. |
| **23** | Cook^(24)^ | 1999 | Australia/ PNG/ China | Both | Estimated and measured amounts (depending on recruitment site): estimated by senior MW/ clinician. Measured blood loss with calibrated measuring jug plus weight of sheets/pads. |
| **24** | Dabbaghi Gale^(25)^ | 2012 | Iran | Not stated | - |
| **25** | Dasuki^(26)^ | 2002 | Indonesia | Not stated | - |
| **26** | Diallo^(27)^ | 2017 | Senegal | Measured | Blood loss collected in a basin 2hrs after delivery, then transferred to graduated measuring jar. |
| **27** | Dutta^(28)^ | 2016 | India | Not stated | - |
| **28** | Eftekhari^(29)^ | 2009 | Iran | Measured | Blood loss in suction bottle measured (minus amniotic fluid), drapes and pads weighed and added to total volume. |
| **29** | Elbohoty^(30)^ | 2016 | Egypt | Measured | Intraop blood loss calculated with: weighed towels + suction bottle contents - amniotic fluid volume. |
| **30** | El-Refaey^(31)^ | 2000 | UK | Visually estimated | “blood loss was estimated subjectively by the attendant midwife or obstetrician.” |
| **31** | Fakour^(32)^ | 2013 | Iran | Not stated | - |
| **32** | Fazel^(33)^ | 2013 | Iran | Not stated | - |
| **33** | Gavilanes^(34)^ | 2016 | Ecuador | Measured | Blood loss was calculated once surgery was completed by measuring blood in the suction apparatus and sterile drapes before irrigation and by evaluating the blood in abdominal swabs and gauzes using a calibrated balance (Zhongshan Camry Electronic Co. Ltd, model EK 4052-E, Guangdong, China). Bleeding volume was estimated by subtraction of amniotic fluid at 30 cc per each centimeter reported by AFI. |
| **34** | Gerstenfeld^(35)^ | 2001 | US | Both | Under buttocks drape- measured in ml, weight of linen and sponges (wet-dry weight), adjusted for weight of amniotic fluid. Also did visual estimation. |
| **35** | Ghafoor^(36)^ | 2022 | Pakistan | Measured | Blood loss estimated by pad test (weighing soaked pads). |
| **36** | Gulmezoglu^(37)^ | 2001 | Many | Measured | Flat bedpan placed under buttocks of women, poured into a standard measuring jar, gauzes were added to jar (these were estimated to result in 10% increase in measurements). |
| **37** | Gupta^(38)^ | 2006 | India | Measured | Linen soiled with blood and amniotic fluid was changed and a calibrated drape (BRASSS-V drape) was placed underneath the buttocks of the parturi- ent to facilitate subsequent blood collection [4]. Preweighed gauzes were used to clean the perineal tears or episiotomy. After one hour the dry weight of the sponges was subtracted from the blood soiled weight. One gram of blood was taken as equiva- lent to one ml and this amount was added to the amount of blood collected in drape to determine to- tal blood loss. |
| **38** | Jain^(39)^ | 2019 | India | Measured | BRASSS drape used, blood-soaked swabs weighed and added to final volume. |
| **39** | Karkanis^(40)^ | 2002 | Canada | N/A | EBL not an outcome of this study. |
| **40** | Kaudel^(41)^ | 2020 | Nepal | Visually estimated | Amount of blood loss was visually estimated at the end of delivery. |
| **41** | Kundodyiwa^(42)^ | 2001 | Zimbabwe | Measured | Fresh disposable incontinence pad placed underneath woman to facilitate further blood collection. Blood expressed from the uterus then measured using a calibrated measuring jug. Amount of blood soiling was determined by weighing the linen saver and sanitary napkins and subtracting their known dry weights. The total measured blood loss was the obtained by adding this amount to the measured blood volume from the jug. |
| **42** | Lokugamage^(43)^ | 2001 | UK | Visually estimated | Blood loss during CS was estimated visually in a standard manner (volume of blood in suction bottle plus soiling of swabs and bed sheets). A record was kept if additional oxytocics used, method of placental removal, presences of abnormal placentation and whether the procedure was difficult or there was marked trauma to surrounding tissues which caused additional blood loss. |
| **43** | Lumbiganon^(44)^ | 1999 | South Africa/ Thailand | Measured | Measured from time of delivery until mother transferred to postnatal ward, collected in standard measuring jar provided by WHO and blood loss measured, linen not weighed but small gauzes put into the measuring jar and included in the measurements together with the blood and clots. |
| **44** | Mishra^(45)^ | 2023 | India | Measured | Sterile kidney dish placed against buttocks, pads and linen weighed. |
| **45** | Mitwaly^(46)^ | 2022 | Egypt | Measured | Towels used in surgery weighed, combined with blood in suction apparatus from surgery. |
| **46** | Modi^(47)^ | 2014 | India | Measured | BRASS-V drapes were applied to measure the amount of blood loss. |
| **47** | Mukta^(48)^ | 2013 | India | Measured | The amount of blood loss (in ml) was estimated by calibrated plastic blood collection drape in which blood was collected after drainage of liquor and delivery of the baby. |
| **48** | Musa^(49)^ | 2015 | Nigeria | Measured | Blood loss at delivery was assessed by the outcome assessor using the gravimetric method [17]. |
| **49** | Najeeb^(50)^ | 2022 | Pakistan | Not adequately stated | “soaked pads were evaluated for blood loss and total blood loss was evaluated” |
| **50** | Nankaly^(51)^ | 2016 | Iran | Not adequately stated | “calculating the total collected blood in suction container and counting the number of blood gases” |
| **51** | Narrey^(52)^ | 2023 | India | Measured | BRASS-V drape used, this blood then transferred to calibrated measuring jug, addition of gauzes and swabs |
| **52** | Nasr^(53)^ | 2009 | Egypt | Visually estimated | “subjective estimation of blood loss by the obstetrician” |
| **53** | Ng^(54)^ | 2004 | Hong Kong | Visually estimated | The amount of blood loss during delivery was assessed by clinical estimation by the attending obstetrician/midwife. Amount of postpartum blood loss is routinely documented in our unit since 1992 as part of the HKCOG and HK Hospital Authority maternity KPI. The amount of blood loss was based on prevailing clinical practice at the time the study was performed. |
| **54** | Oboro^(55)^ | 2003 | Nigeria | Visually estimated | “the delivering obstetrician estimated blood loss” |
| **55** | Othman^(56)^ | 2016 | Egypt | Measured | Adding volume of blood in suction bottle with the weight of blood-soaked sponges and the collection drape (collection drape used for EBL 2hrs post op). |
| **56** | Owonikoko^(57)^ | 2011 | Nigeria | Visually estimated | Multiple assessments of blood loss taken, the final value was the average. Estimation by obstetrician, estimation by anaesthetist (aware of pt allocation), scrub nurse weighed drapes and gauze and added suction volume. |
| **57** | Pakniat^(58)^ | 2015 | Iran | Not adequately described | Volume of blood in the suction bottle and blood-soaked sponges was measured. |
| **58** | Parsons^(59)^ | 2006 | Canada | Visually estimated | Blood loss was estimated by the nurse midwife of health officer and recorded but was not objectively measured |
| **59** | Parsons^(60)^ | 2007 | Canada | Visually estimated | A subjective estimate of blood loss was recorded |
| **60** | Patil^(61)^ | 2023 | India | Measured | Blood clots were collected and weighed; blood loss was calculated accordingly (1gm of blood clot = 4ml blood loss). |
| **61** | Penaranda^(62)^ | 2002 | Colombia | Not stated | “Bleeding was quantified from clamping of the umbilical cord until one hour after delivery.” |
| **62** | Perez Rumbos^(63)^ | 2017 | Venezuela | Measured | Collected in a calibrated plastic container. All the gauzes used were collected, weighed and added to the value of the plastic container. |
| **63** | Rajaei^(64)^ | 2014 | Iran | Measured | Weighed pads only. |
| **64** | Rawat^(65)^ | 2021 | India | Measured | Kidney tray beneath buttocks and weighing soaked pads. |
| **65** | Roy^(66)^ | 2017 | India | Not stated | “estimation of average blood loss … was measured.” |
| **66** | Sadiq^(67)^ | 2011 | Nigeria | Visually estimated | Calibrated kidney dishes, measured to nearest mL. |
| **67** | Satyajit^(68)^ | 2017 | India | Measured | BRASS-V drape measured blood loss + weight of blood clots, addition of blood soaked swabs. |
| **68** | Shady^(69)^ | 2017 | Egypt | Measured | Blood collected in a drape + weighed pads. |
| **69** | Shah^(70)^ | 2021 | Pakistan | Measured | Surgical gauzes were weighed, and the difference noted. |
| **70** | Shaheen^(71)^ | 2019 | Pakistan | Visually estimated | A pictoral visual assessment chart was used to aid accurate assessment. |
| **71** | Shrestha^(72)^ | 2011 | Nepal | Measured | Preweighed calibrated sterile drapes + weight of pads. |
| **72** | Singh^(73)^ | 2009 | India | Measured | Linen saver sheet with bag for collecting blood, weighed and added to weight of pads. |
| **73** | Snehalata^(74)^ | 2023 | India | Not adequately stated | Quantified in calibrated glass container. |
| **74** | Sultana^(75)^ | 2007 | Bangladesh | Visually estimated | Estimated on approximate basis by the delivering physician after collecting blood within a plastic bowl. |
| **75** | Tewatia^(76)^ | 2014 | India | Measured | Calibrated sheet + pads used in weight but blood loss while repairing episiotomy not included. |
| **76** | Vagge^(77)^ | 2014 | India | Not described | “Secondary outcomes were taken as the amount of blood loss …” |
| **77** | Vimala^(78)^ | 2006 | India | Measured | Volume of blood in the suction bottle was measured, blood-soaked sponges and linen savers were weighed and known dry weight subtracted and added to volume from the suction bottle. |
| **78** | Walley^(79)^ | 2000 | Canada | Visually estimated | Visually estimated by attending physician/midwife. |
| **79** | Zachariah^(80)^ | 2006 | India | Measured | Large sterile bag placed under woman, blood from bag transferred to measuring jar plus gauzes used. |

**Appendix I:** List of RCTs in full text screening that were excluded

| Year | Trial identifier/ Reference to publication | Reason for exclusion |
| --- | --- | --- |
| 2011 | NCT01508429 | Wrong intervention (misoprostol vs placebo) |
| 2012 | NCT01710566 | Wrong study design (cluster RCT) |
| 2012 | NCT01713153 | Wrong study design (cluster RCT) |
| 2019 | NCT03870503 | Wrong intervention (misoprostol + oxytocin vs oxytocin) |
| 2011 | NCT01487278 | Wrong study design (cluster RCT) |
| 2014 | NCT02277041 | Wrong intervention (carbetocin + oxytocin vs oxytocin) |
| 2014 | NCT02304042 | Wrong intervention (carbetocin + oxytocin vs oxytocin) |
| 2018 | Akhter P, Pal SN, Begum S. Comparison between Carbetocin and Oxytocin in Active Management of 3rd Stage of Labour in Preventing Post Partum Hemorrhage. Mymensingh Med J. 2018;27(4):793-7. | Wrong intervention (carbetocin vs oxytocin) |
| 2019 | Maged AM, Fawzi T, Shalaby MA, Samy A, Rabee MA, Ali AS, et al. A randomized controlled trial of the safety and efficacy of preoperative rectal misoprostol for prevention of intraoperative and postoperative blood loss at elective cesarean delivery. Int J Gynaecol Obstet. 2019;147(1):102-7. | Wrong intervention (misoprostol vs misoprostol) |
| 2013 | Safety and Efficacy of Misoprostol Versus Oxytocin for Prevention of Post Partum Hemorrhage | Duplicate |
| 2016 | Oxytocin via Uniject (a prefilled single‐use injection) versus oral misoprostol for prevention of postpartum haemorrhage at the community level: a cluster‐randomised controlled trial. Lancet. Global Health 2016;4(1):e37‐44. | Wrong study design (cluster RCT) |
| 2006 | Lapaire O, Schneider MC, Stotz M, Surbek DV, Holzgreve W, Hoesli IM. Oral misoprostol vs. intravenous oxytocin in reducing blood loss after emergency cesarean delivery. International Journal of Gynecology & Obstetrics 2006;95(1):2‐7. - PubMed | Wrong intervention (Misoprostol + oxytocin vs oxytocin) |
| 2015 | [Rozenberg P, Quibel T, Ghout I, Salomon L, Bussiere L, Goffinet F. Active management of the third stage of labor with routine oxytocin and misoprostol for the prevention of postpartum hemorrhage: a randomized controlled trial. American Journal of Obstetrics and Gynecology 2015;212(1 Suppl 1):S18.](https://pubmed.ncbi.nlm.nih.gov/27607864/) | Wrong intervention (Misoprostol + oxytocin vs oxytocin) |

**Appendix J:** Funnel plots for the primary outcome PPH≥500mL

|  |  |
| --- | --- |
| Figure A: RCTs participating in IPD-MA (n=6) | Figure B: All RCTs participating and non-participating (n=43) |

**Appendix K:** Funnel plots for the primary outcome PPH≥1000mL

|  |  |
| --- | --- |
| Figure A: RCTs participating in IPD-MA (n=3) | Figure B: All RCTs participating and non-participating (n=34) |
